# Bone–Screw–Force Interactions in Palatal Orthodontic Mini-Implants: A Scoping Review and Decision Framework for Primary Stability and Biomechanically Driven Site Selection

**DOI:** 10.3390/jfb17080360

**Published:** 2026-07-26

**Authors:** Mahmoud Elsaafin, Alexandra Mihaela Stoica, Marius Mariș, Adina Simona Coșarcă, Liana Bereșescu, Ahmed Elsaafin, Mariana Păcurar, Valeriu Mihai But

**Affiliations:** 1 Department of Orthodontics, Faculty of Dental Medicine, George Emil Palade University of Medicine, Pharmacy, Science, and Technology of Târgu Mureș, 38 Gh. Marinescu Str., 540142 Târgu Mureș, Romania; nabil.elsaafinmahmoud@umfst.ro (M.E.); mariana.pacurar@umfst.ro (M.P.); 2 Department of Odontology and Oral Pathology, George Emil Palade University of Medicine, Pharmacy, Science, and Technology of Târgu Mureș, 540139 Târgu Mureș, Romania; 3 Department of Orthodontics and Dentofacial Orthopedics, Faculty of Dental Medicine, Titu Maiorescu University, 67A Gheorghe Petrașcu Street, 031593 Bucharest, Romania; marius.maris@prof.utm.ro; 4 Department of Oral and Maxillofacial Surgery, Faculty of Dental Medicine, George Emil Palade University of Medicine, Pharmacy, Science, and Technology of Târgu Mureș, 38 Gheorghe Marinescu Street, 540139 Târgu Mureș, Romania; adina.cosarca@umfst.ro; 5 Department of Preventive and Community Dentistry, Faculty of Dental Medicine, George Emil Palade University of Medicine, Pharmacy, Science, and Technology of Târgu Mureș, 38 Gheorghe Marinescu Street, 540139 Târgu Mureș, Romania; liana.beresescu@umfst.ro; 6 Department of Plastic Surgery and Reconstructive Microsurgery, Târgu Mureș County Emergency Clinical Hospital, 50 Gheorghe Marinescu Street, 540136 Târgu Mureș, Romania; amsaafin@hotmail.com; 7 Department of Medicine-Psycho-Neuroscience and Recovery, Faculty of Medicine and Pharmacy, University of Oradea, 410073 Oradea, Romania; dr.butvaleriu@gmail.com

**Keywords:** palatal mini-implants, orthodontic miniscrews, temporary anchorage devices, scoping review, primary stability, insertion torque, cortical bone, mucosal thickness, MARPE, digital planning, biomaterials, surface treatment, failure mode

## Abstract

Background: Palatal orthodontic mini-implants are increasingly used for sagittal, vertical, and transverse mechanics, including molar distalization, mesialization, posterior intrusion, impacted tooth traction, and miniscrew-assisted rapid palatal expansion. Yet site selection is often discussed as if anatomical bone availability were the dominant determinant of performance. Objective: This scoping review maps direct palatal evidence and supporting mechanistic evidence on how palatal substrate, miniscrew design, insertion protocol, digital planning, biomaterial surface, and biomechanical loading interact to determine primary stability, loaded stability, survival, and failure risk. Methods: Records were searched in PubMed/MEDLINE, Scopus and Web of Science Core Collection and organized into two evidence streams, as follows: P1 direct palatal evidence and P2 supporting mechanistic/biomaterials evidence. Data were charted using a Bone–Screw–Force framework. Results: The evidence indicates that anterior palatal and anterior paramedian sites are usually favorable for routine anchorage, but posterior sites, PPSAIS, and MARPE locations may warrant patient-specific three-dimensional assessment when clinical and conventional radiographic evaluation is insufficient. Primary stability emerges from cortical thickness, trabecular quality, effective intraosseous length, miniscrew diameter, thread design, insertion angle, pilot-hole protocol, torque, force magnitude, and biological response. Digital workflows are best interpreted as trajectory-control technologies rather than as convenience tools. Biomaterial and surface evidence remains promising but insufficiently connected to palatal-specific outcomes. Conclusions: Palatal miniscrew performance should be reframed as an interface problem rather than a site-only problem. A Bone–Screw–Force framework can support biomechanically driven site selection while identifying where quantitative evidence remains insufficient.

## 1. Introduction

Temporary anchorage devices (TADs) have changed orthodontic biomechanics by reducing dependence on reciprocal dental anchorage and patient compliance. Among extra-alveolar regions, the palate has become especially attractive because selected anterior and paramedian areas combine keratinized mucosa, relative distance from interradicular root constraints, and access to rigid appliance frameworks for sagittal, vertical, and transverse mechanics. Early clinical protocols, T-zone recommendations, and anatomical guidelines established the anterior palate as a reliable anchorage region [1,2,3]. Subsequent CBCT mapping showed that this reliability is site-specific rather than uniform across the palate [4,5].

The anatomical component of palatal anchorage is heterogeneous. Median, paramedian, posterior, and supra-alveolar regions differ in total bone depth, cortical thickness, cancellous density, mucosal thickness, nasopalatine canal proximity, nasal-floor relationship, and root-related risk. Classical CT and CBCT studies described the general availability of anterior palatal bone [6,7,8]. More recent investigations refined this map by adding age, sex, skeletal maturation, palatal morphology, mucosal thickness, and posterior site variation [9,10,11,12,13].

The screw component is equally important. Diameter, length, effective intraosseous engagement, thread pitch and depth, taper, material, surface state, insertion torque, pilot-hole preparation, and insertion angle can alter primary stability, cortical microdamage, fracture risk, and loaded displacement. Mechanistic studies on bone quality and intrabony length indicate that stability is partly substrate-dependent [14,15,16]. Studies on implant design, cortical damage, insertion torque, and site preparation further suggest that a palatal miniscrew should be interpreted as a temporary functional biomaterial device rather than as a coordinate placed into bone [17,18,19,20].

The force component completes the interface. Routine indirect anchorage, molar distalization, posterior intrusion, impacted-tooth traction, and MARPE expansion do not impose the same magnitude, line of action, moment system, timing, or appliance rigidity. High-load transverse expansion behaves as a stress test of the screw–bone–appliance complex [21,22,23,24]. Sagittal and vertical mechanics may be limited more by vector control, connector stiffness, soft-tissue tolerance, or appliance deformation than by absolute bone thickness alone [25,26,27,28].

Digital planning and biomaterials research add two additional dimensions. CBCT/DICOM-STL matching, CAD/CAM insertion guides, dynamic navigation, and one-visit appliance workflows aim to reduce the difference between planned and achieved screw trajectory [29,30,31,32]. At the same time, studies of titanium alloys, stainless steel, zirconia, surface treatments, corrosion, degradation, removal torque, and biological response show that clinical stability also depends on device-level properties and host response [33,34,35].

### 1.1. Why a Site-Only Review Would Be Insufficient

A site-only review would rank palatal regions by average bone thickness and would then conclude that the anterior palate is generally favorable. That conclusion is useful but incomplete. Palatal site selection must distinguish between nominal bone availability and clinically usable engagement. Thick mucosa can reduce effective intraosseous screw length; the incisive canal and roots can limit safe trajectories; posterior areas can be useful but anatomically variable; and the same site can have different implications depending on screw length, collar design, and force vector [4,9,10,11,36,37,38].

### 1.2. Intended Contribution of This Review

The intended contribution of this review is not to repeat that the anterior palate is favorable. Instead, it is to define when that principle is sufficient and when it is not. Routine anterior palatal anchorage can often be planned from established safe-zone principles, but adult MARPE, posterior anchorage, PPSAIS placement, special anatomy, high/narrow palates, cleft-related anatomy, and high-load vectors require more rigorous assessment of bone, mucosa, screw geometry, and force demand [2,3,9,21,39,40,41].

Finally, the proposed decision framework should be interpreted as a synthesis tool rather than a rigid treatment protocol. It guides the clinician to identify the objective, estimate load level, assess anatomical risk, select the site, match screw dimensions and insertion strategy, decide whether digital planning is required, and anticipate the most likely failure mode. This sequence reflects the way palatal mini-implants function clinically: as components of a biomechanical and biological interface rather than as isolated anchorage pins [14,27,28,29,30,32,42,43].

## 2. Materials and Methods

### 2.1. Review Design and Reporting Framework

This work was designed as a scoping review because the objective was to map, categorize, and synthesize a heterogeneous evidence base rather than to estimate a single pooled effect. The reporting logic followed PRISMA-ScR and contemporary methodological guidance for scoping reviews; PRISMA 2020 was used to structure the search-flow reporting, while narrative-review quality concepts were used only as secondary safeguards for clarity and reasoning [44,45,46,47,48,49,50].

The review protocol was not prospectively registered. To improve transparency, the protocol and supporting materials were retrospectively registered on the Open Science Framework on 17 July 2026 (https://osf.io/5bcxu; accessed on 24 July 2026). Registration occurred after data charting had commenced and is therefore explicitly reported as retrospective.

The central review question was as follows: How do palatal anatomical substrate, orthodontic mini-implant design, insertion-related variables, and biomechanical loading interact to influence primary stability, loaded stability, survival, failure modes, and site selection of palatal orthodontic mini-implants? The PCC framework is summarized in Table 1.

### 2.2. Information Sources, Search Blocks, and Eligibility

PubMed/MEDLINE, Scopus and Web of Science Core Collection were searched from database inception to 26 May 2026, with no date or language restrictions applied during the search. The complete database-specific strategies, including exact query strings, field tags, Boolean operators, applied limits, search date, and yield for each database, are provided in Supplementary Table S11. Records were exported from PubMed/MEDLINE as NBIB files and from Scopus and Web of Science Core Collection as RIS/CSV citation files. Deduplication was performed using a custom Python 3.12.13-based workflow (Python Software Foundation, Wilmington, DE, USA; https://www.python.org/) applied to exported NBIB/RIS/CSV records. Duplicate detection prioritized exact DOI matches, followed by PMID matches, normalized-title matches, and title-year-first-author matching for records without DOI or PMID. Ambiguous exact-title groups were retained for manual review.

Detailed eligibility logic and full-text decision rules are provided in Supplementary Table S1.

### 2.3. Evidence Streams and Data Charting

To prevent a purely narrative synthesis, the evidence was separated into two streams. P1 contained direct palatal evidence, including CBCT anatomy, palatal survival, MARPE, palatal digital workflows, and palatal clinical applications. P2 contained supporting mechanistic evidence, including insertion torque, thread design, material, coating, microdamage, fracture, and biological-response studies. Supporting evidence from non-palatal or interradicular studies was used only to interpret general screw–bone mechanics and was not treated as direct proof for palatal site selection. Two reviewers independently assessed the records at both stages against the eligibility criteria. Title and abstract screening was conducted between 29 May and 7 June 2026, followed by full-text assessment between 8 and 20 June 2026. Screening decisions were recorded in structured Microsoft Excel for Microsoft 365 workbooks (Microsoft Corporation, Redmond, WA, USA; https://www.microsoft.com/microsoft-365/excel, accessed on 24 July 2026). Disagreements were resolved through discussion and consensus; no third reviewer was required.

Detailed evidence-stream definitions and synthesis functions are provided in Supplementary Table S2.

### 2.4. Data Charting and Extraction Variables

Data charting was organized around variables that could influence decision making. For anatomical papers, the core fields were study design, sample, age group, imaging method, reference grid, palatal region, bone depth, cortical thickness, mucosal thickness, canal/root relationship, and stated safe-zone conclusion. For MARPE papers, the charting fields included age, maturation status, appliance type, number and location of screws, mono- or bicortical engagement when reported, activation protocol, skeletal response, dentoalveolar effects, complications, and follow-up.

For stability and biomaterials papers, charting focused on material, diameter, length, thread, taper, head/collar design, surface treatment, insertion protocol, pilot-hole diameter, insertion angle, torque, removal torque, pull-out force, fracture torque, RFA/ISQ, bone-miniscrew contact, microdamage, and biological response. These variables were intentionally preserved even when the paper was not palatal-specific, because they help explain why a palatal screw may or may not perform well once anatomical feasibility has been established.

For digital workflow papers, the most important extraction fields were planning method, data sources, guide support, manufacturing technique, navigation type, planned-versus-achieved deviation, angular error, coronal/apical deviation, laboratory versus clinical error, and whether appliance delivery was simultaneous with insertion. These variables are more informative than a generic statement that digital planning improves accuracy.

Data charting and extraction were performed independently by two authors using a standardized Microsoft Excel for Microsoft 365 workbook (Microsoft Corporation, Redmond, WA, USA; https://www.microsoft.com/microsoft-365/excel, accessed on 24 July 2026). The independently charted datasets were compared, and discrepancies were resolved through discussion and consensus.

A record-level inventory is provided in Supplementary Data S2. The workbook preserves the separate P1 (*n* = 596) and P2 (*n* = 688) record structures and, where available, reports the screening identifier, bibliographic citation, DOI, evidence role, mapped Bone–Screw–Force domains, and destination in the synthesis.

All evidence sources retained in the final map were eligible for structured data charting. Numerical values were recorded when they addressed a prespecified Bone–Screw–Force variable and were directly traceable to the source. No fixed numerical-extraction subset or 120-record sampling stratum was used.

### 2.5. Evidence Synthesis and Tabular Synthesis Strategy

Evidence was synthesized in layered tables rather than in a purely narrative format. Direct palatal evidence was used for site selection, safety, and clinical indication mapping, whereas supporting mechanistic evidence was used to interpret the screw–bone interface, insertion mechanics, material behavior, and biological response. This separation was maintained throughout the Results so that direct clinical recommendations were not inferred from indirect laboratory, animal, or finite-element evidence.

Study-level comparison was organized in the Results through evidence-family tables. This organization is appropriate for a scoping review because readers should be able to see how evidence was mapped, which variables were extracted, and where the evidence is direct, indirect, or underreported.

Evidence directness was preserved throughout the synthesis. Direct human palatal clinical studies informed clinical outcomes; human palatal imaging and anatomical studies informed anatomical feasibility; and non-palatal clinical comparators, finite-element, animal, cadaveric, in vitro, and synthetic-bone studies were used only for mechanistic interpretation or hypothesis generation. Heterogeneous designs were not pooled or treated as having equivalent clinical weight, and no clinical site-selection recommendation was based solely on indirect evidence.

The Bone–Screw–Force framework is an author-derived conceptual synthesis developed from the mapped evidence. It has not undergone formal validation and is not intended as a clinical prediction rule or prescriptive decision tool.

## 3. Results

### 3.1. Search Output and Evidence Architecture

**Figure 1:**
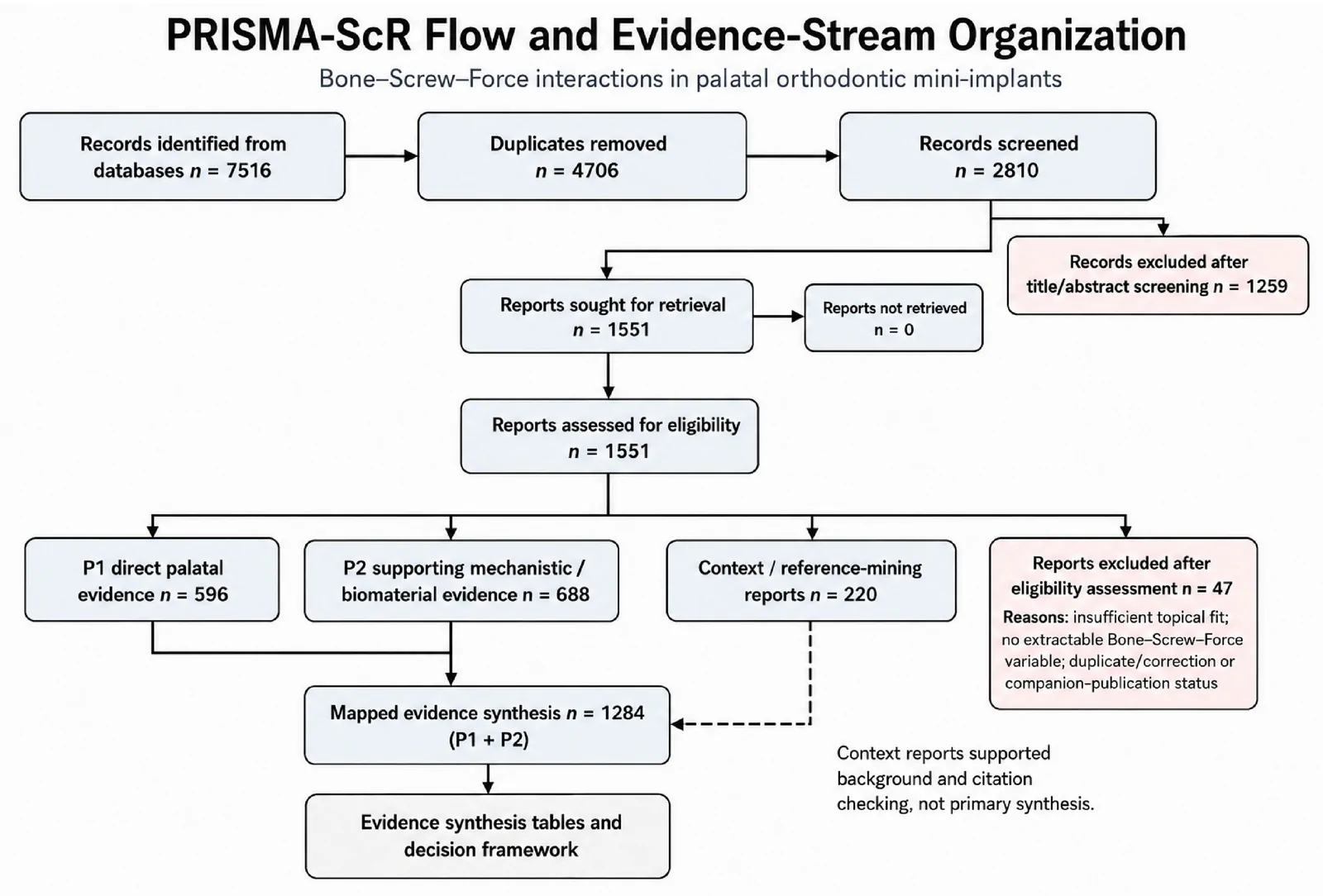
PRISMA-ScR flow diagram showing records identified, screened, sought for retrieval, assessed for eligibility, excluded with reasons, and retained as evidence sources. The flow diagram was prepared by the authors using Seedream 5. The dashed arrow denotes contextual/background support only; context/reference-mining reports were not included in the primary mapped evidence synthesis.

The searches yielded 7516 records, as follows: 2022 from PubMed/MEDLINE, 2698 from Scopus, and 2796 from Web of Science Core Collection. After removal of 4706 duplicates, 2810 records underwent title/abstract screening, of which 1259 were excluded. A total of 1551 reports were sought and retrieved for full-text assessment. Forty-seven reports were excluded after eligibility assessment, and 220 context or reference-mining reports were retained solely for background and citation checking but were not included in the mapped evidence synthesis. The final evidence map comprised 1284 sources, as follows: 596 in the direct palatal evidence stream (P1) and 688 in the supporting mechanistic/biomaterials stream (P2). These counts reconcile to the complete record-level inventory provided in Supplementary Data S2. The evidence-selection flow is shown in Figure 1.

### 3.2. Bone Domain: Palatal Anatomy, Landmarks, and Patient-Related Modifiers

The direct palatal evidence shows that the anterior palate is favorable in many patients, but the pattern is more nuanced than a simple anterior-versus-posterior statement. Studies differ in grid origin, mediolateral offset, anteroposterior reference, age range, sex distribution, skeletal pattern, growth status, and whether mucosal thickness is assessed. For this reason, anatomical evidence and the site-specific palatal matrix should be interpreted together, as follows: the first maps the substrate and landmarks, while the second translates those landmarks into clinically usable anchorage regions.

Table 2 groups anatomical studies by the type of clinical landmark or anatomical question they address, rather than listing each study individually. This organization distinguishes anterior-palatal safe-zone mapping, posterior and supra-alveolar corridors, appliance-specific palatal sites, canal/root safety, and patient-related modifiers such as age, skeletal maturation, facial pattern, palatal morphology, population, sex, and ethnicity. Patient-related anatomical modifiers were treated as a distinct interpretive layer. Age and skeletal maturation influence sutural biology and palatal bone characteristics; facial pattern and palatal morphology modify available bone and access; population, sex, and ethnicity influence dimensional averages; and special anatomy, including cleft or syndromic patients, requires individualized imaging rather than transfer of generic safe-zone values.

A key implication of Table 2 is that anatomical recommendations must be expressed as probability-weighted and site-specific, not as universal rules. The anterior paramedian palate and T-zone are often favorable, but this does not automatically translate to every patient, every screw dimension, or every force system. Posterior anchorage, PPSAIS, and MARPE screw positions may warrant patient-specific three-dimensional assessment when clinical and conventional radiographic evaluation cannot adequately determine bone availability, anatomical risk, cortical engagement, or insertion trajectory.

### 3.3. Soft Tissue, Effective Intraosseous Length, and Safety Landmarks

Nominal screw length should not be equated with bone-engaged thread length. Effective intraosseous engagement cannot be estimated by simply subtracting mucosal thickness from nominal screw length; it also depends on the threaded-segment length, transmucosal collar, local bone-surface geometry, insertion angle and trajectory, and intended mono- or bicortical engagement. The main soft-tissue and safety modifiers are summarized in Table 3.

### 3.4. Site-Specific Palatal Matrix: Clinical Application of Anatomical Evidence

Table 4 combines the anatomical evidence from Section 3.2 with appliance-specific palatal anchorage requirements. Distalizer-specific sites, mesializer-supported palatal anchorage, expansion frameworks, and MARPE screw zones should be interpreted as application-dependent site categories, not as interchangeable anatomical coordinates.

### 3.5. MARPE and Other High-Load Transverse Systems

MARPE studies were separated according to appliance architecture rather than pooled as a single expansion category. The mapped evidence included conventional tooth-bone-borne MARPE designs, named Maxillary Skeletal Expander appliances when explicitly reported in included studies, pure bone-borne or hybrid expanders, and customized 3D-printed expanders. This distinction is important because tooth support, skeletal support, screw number, screw position, appliance rigidity, activation protocol, and cortical engagement influence force transmission and reported success. Activation protocols should also be reported explicitly, usually as turns per day or millimeters of expansion per day, rather than being converted into the same force units used for distalization or intrusion mechanics. Additional MARPE-related studies mapped outcomes that were not used as primary determinants of palatal site selection but remain relevant to interpreting the broader biological and clinical effects of high-load expansion, including airway and nasal-cavity changes, circummaxillary suture width, expansion efficiency, periodontal outcomes, root resorption, customized 3D-printed MARPE appliances, and activation protocols [114,115,116,117,118,119,120,121,122,123,124,125,126,127,128,129]. The mapped high-load MARPE evidence is summarized in Table 5.

### 3.6. Force Domain: Transverse Expansion, Sagittal Distalization/Mesialization, Vertical Intrusion, and Traction Mechanics

Palatal mini-implant site selection should be linked to the intended force system. Transverse expansion is a high-load orthopedic application in which activation rate, appliance rigidity, and cortical engagement are central. Molar distalization and mesialization mainly require sagittal force delivery and moment control. Posterior intrusion requires vertical force vectors that minimize buccal-palatal tipping, whereas impacted canine traction and anterior retraction require controlled directional force delivery. For continuous orthodontic mechanics, force magnitude is commonly reported in grams or newtons per side; by contrast, expansion protocols are reported as screw turns per day, quarter-turns per day, or millimeters of expansion. These load variables should not be pooled.

A detailed clinical-force application inventory is provided in Supplementary Table S3; the decision-level synthesis is retained in the integrated clinical decision matrix.

### 3.7. Digital Planning and Guided Insertion

In this review, digital planning studies were interpreted as evidence on planned-versus-achieved transfer accuracy: how accurately a virtual mini-implant position can be transferred to the patient. The mapped studies included static 3D-printed guides, CAD/CAM one-visit workflows, dynamic navigation or mixed-reality systems, lateral-cephalogram-plus-intraoral-scan workflows, and studies measuring laboratory, clinical, coronal, apical, or angular deviations between the planned and achieved screw position. The digital-planning evidence map is presented in Table 6.

**Figure 2:**
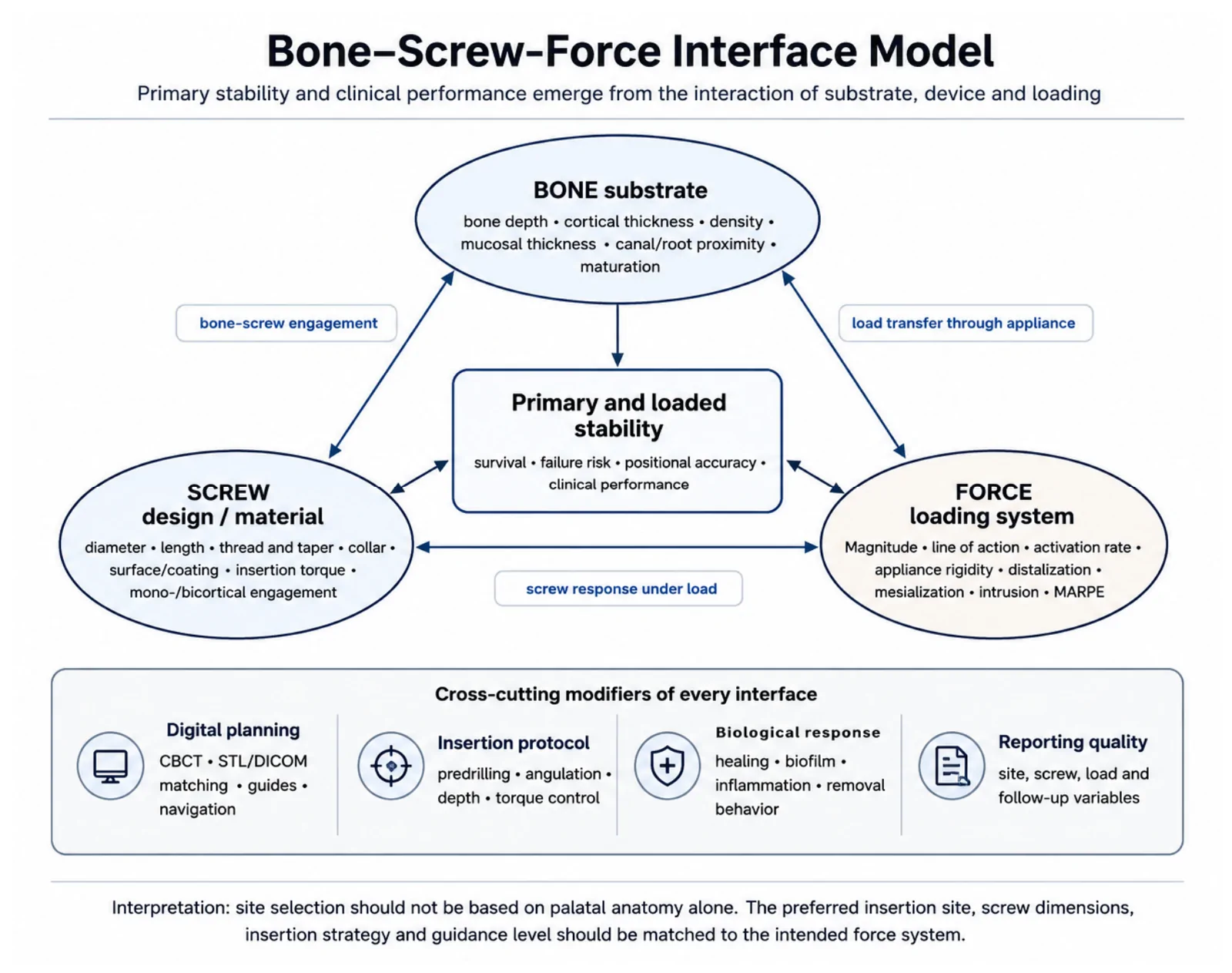
Author-derived Bone–Screw–Force interface model. Palatal mini-implant performance is conceptualized as the interaction of substrate, device, and loading, with digital planning, insertion protocol, biological response, and reporting quality as modifiers. The figure was prepared using Seedream 5; the conceptual design, scientific content, labels, and final verification were performed by the authors.

**Figure 3:**
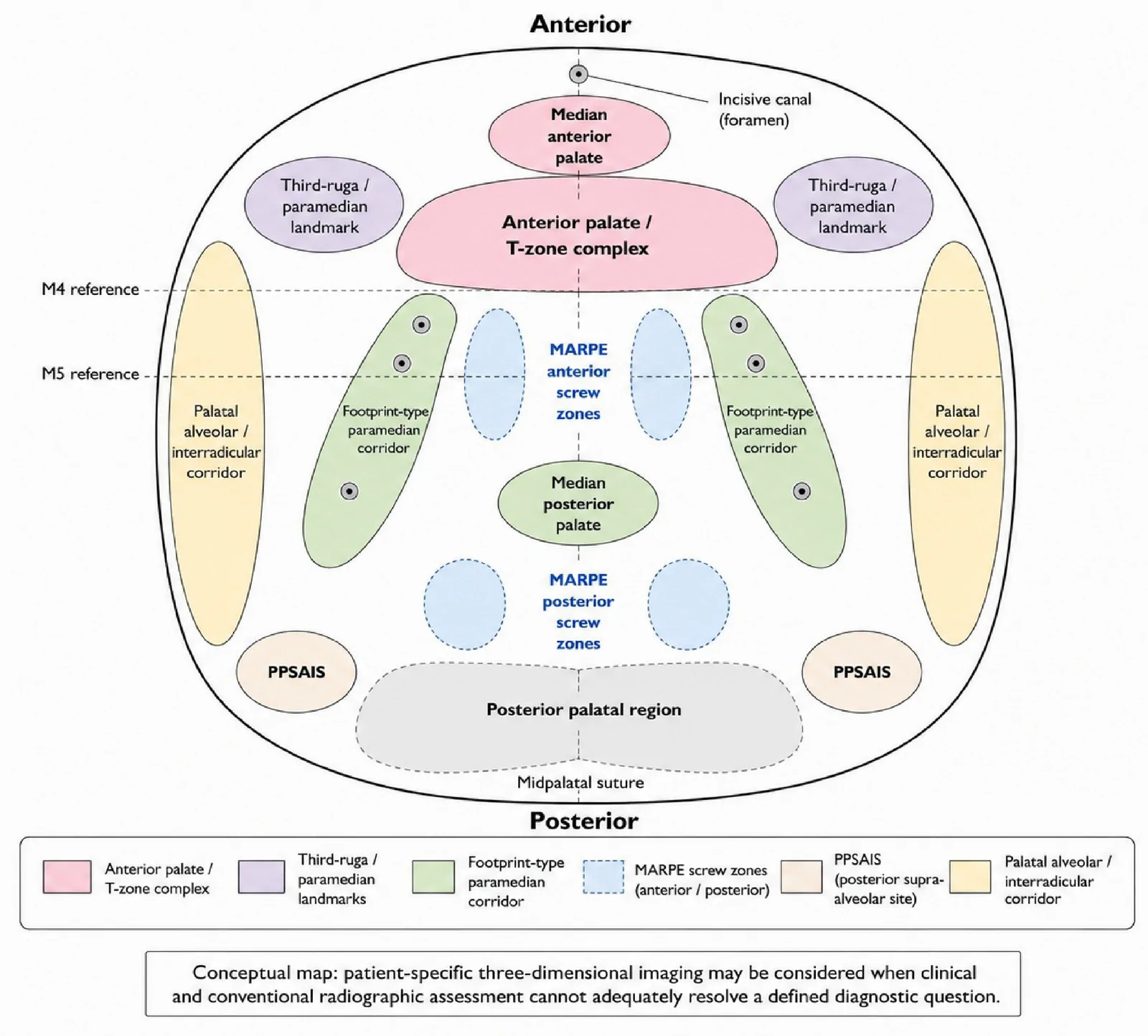
Author-derived schematic map of palatal site categories. The diagram is conceptual and is not a universal safe-zone prescription. Patient-specific three-dimensional imaging may be considered when a defined diagnostic question cannot be resolved adequately by clinical and conventional radiographic assessment. The figure was prepared using Seedream 5; the conceptual design, scientific content, labels, and final verification were performed by the authors. Gray shading denotes the broader posterior palatal region as an anatomical reference and does not represent a validated or universally safe insertion zone.

**Figure 4:**
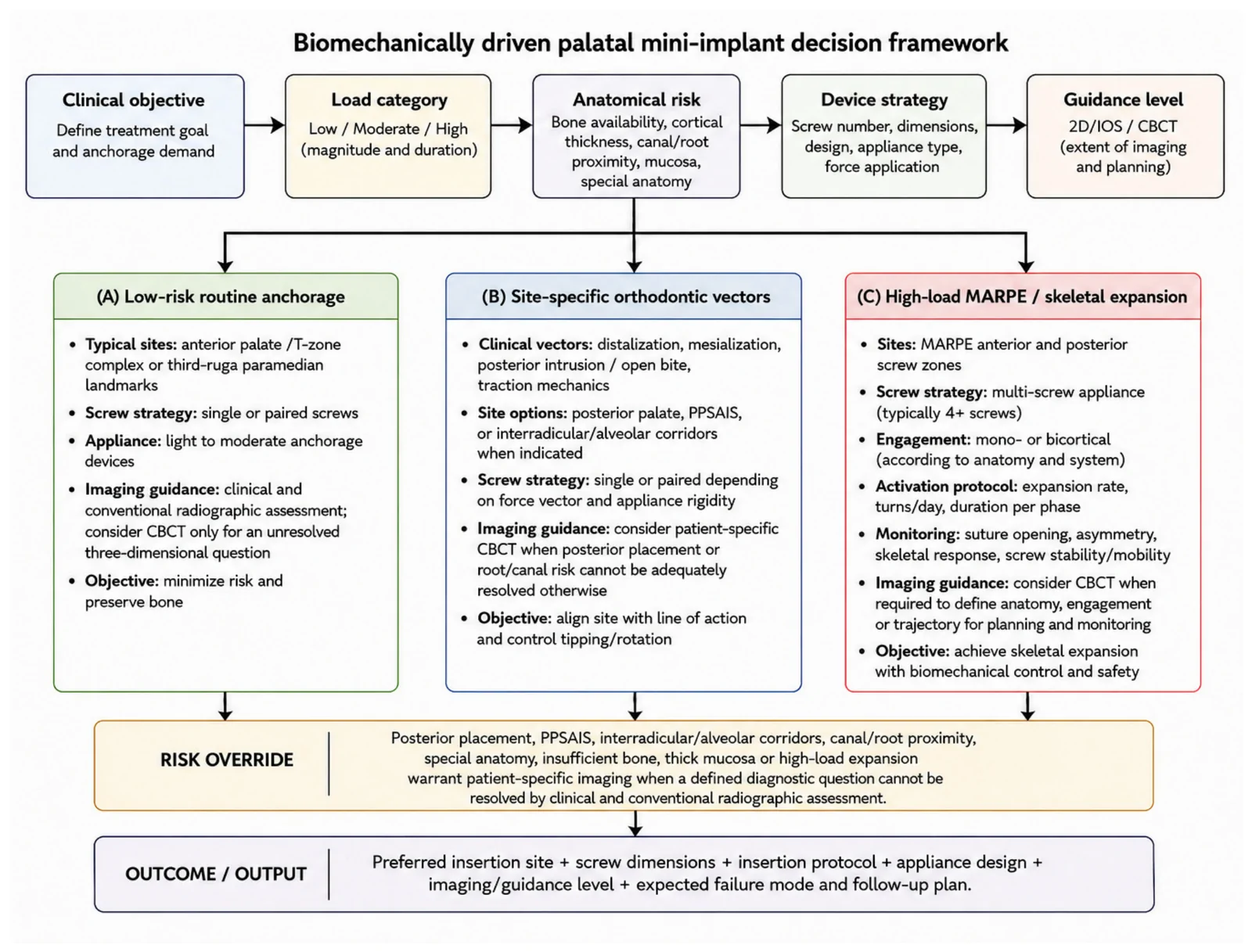
Author-derived, unvalidated Bone–Screw–Force framework linking clinical objective, load, anatomy, device strategy, and guidance. It is a conceptual synthesis rather than a clinical prediction rule or prescriptive decision tool; imaging should be justified by a patient-specific diagnostic question. The figure was prepared using Seedream 5; the conceptual design, scientific content, labels, and final verification were performed by the authors. Colors are used solely to distinguish framework stages and clinical branches; they do not represent validated risk grades or levels of evidence.

**Figure 5:**
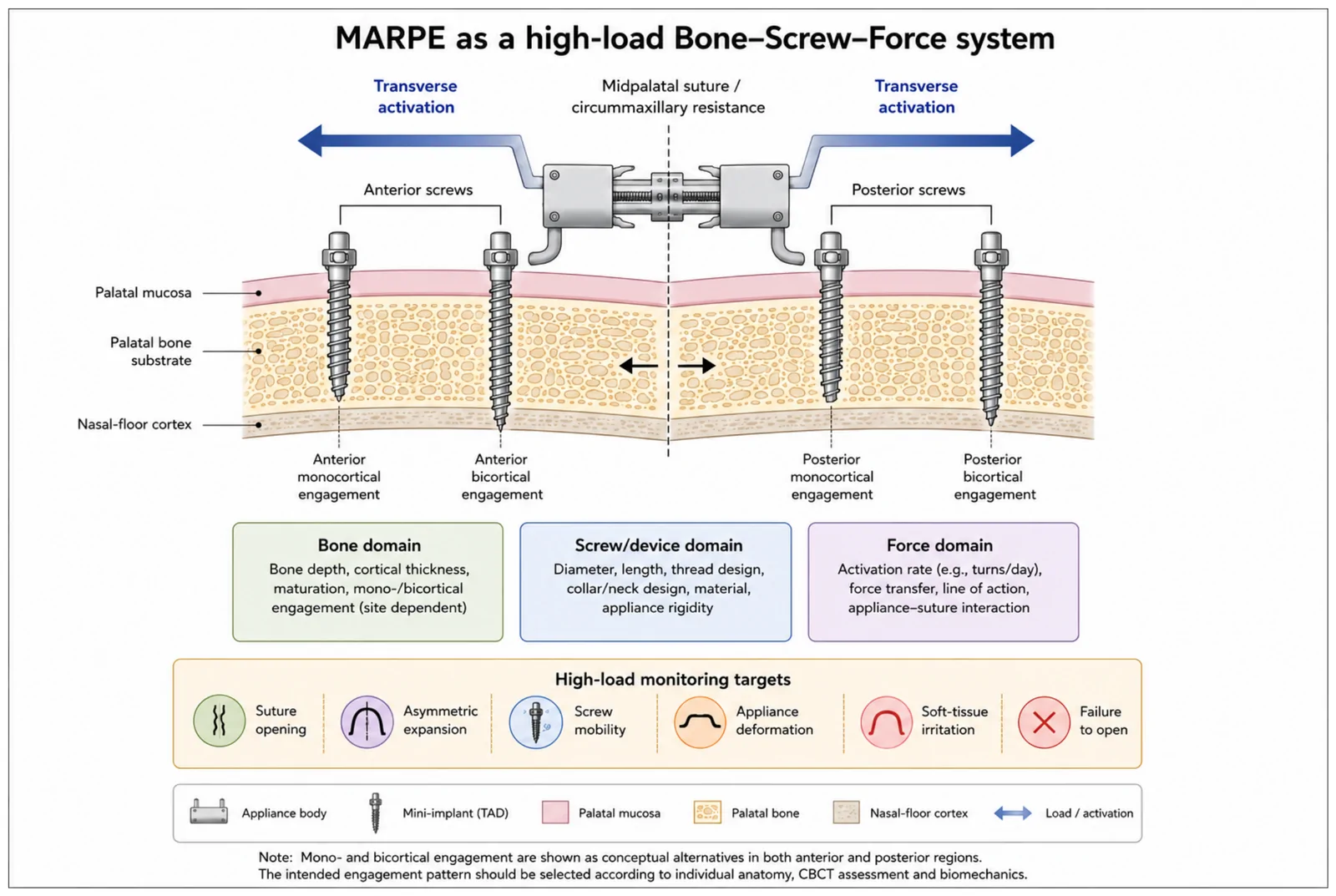
Author-derived conceptual representation of MARPE as a high-load Bone–Screw–Force system. Appliance rigidity, activation, screw position and engagement, palatal substrate, maturation, and biological response jointly influence performance; imaging should be justified by the patient-specific diagnostic question. The figure was prepared using Seedream 5; the conceptual design, scientific content, labels, and final verification were performed by the authors.

The detailed force-application inventory in Supplementary Table S3 further maps palatal and supporting skeletal anchorage evidence for molar distalization, posterior intrusion, aligner-assisted mechanics, open-bite correction, expansion-related mechanics, and Class III or protraction protocols [198,199,200,201,202,203,204,205,206,207,208,209,210,211,212,213,214,215,216,217,218,219,220,221,222,223,224,225,226,227,228,229,230,231,232,233,234,235,236,237,238,239,240,241,242,243,244,245,246]. The mapped evidence is synthesized visually in Figure 2 (interface model), Figure 3 (site categories), Figure 4 (decision framework), and Figure 5 (MARPE high-load system).

### 3.8. Screw Domain: Primary Stability, Insertion Protocol, and Loaded Stability

The mechanistic literature demonstrates that primary stability is not a single measurement. Insertion torque, resonance frequency, pull-out strength, bone–miniscrew contact, removal torque, displacement under load, and failure under cyclic or immediate loading each capture different aspects of the interface. A site with adequate bone may still fail if the screw geometry, pilot-hole strategy, insertion angle, or loading protocol is poorly matched to the substrate.

Because miniscrew diameter differs markedly between interradicular, anterior palatal, posterior palatal, and MARPE applications, this review did not calculate a single pooled average diameter. Instead, diameter was interpreted according to anatomical site and biomechanical demand. Palatal mini-implants are often wider and longer than many interradicular buccal miniscrews, and MARPE screws should be charted separately because their engagement requirements differ from routine anchorage. Wider screws may improve fracture resistance and anchorage stiffness, but their use is constrained by mucosal thickness, canal/root proximity, and available bone volume. These determinants are summarized in Table 7.

A more detailed inventory of supporting primary-stability evidence is provided in Supplementary Table S5, including studies on thread depth and pitch, insertion and removal torque, acid-etched or anodized surfaces, self-drilling and self-tapping protocols, root proximity, fracture resistance, cortical microdamage, dual-thread designs, and pull-out strength [303,330,331,332,333,334,335,336,337,338,339,340,341,342,343,344,345,346,347,348,349,350,351,352,353,354,355,356,357,358,359,360,361,362,363,364,365].

### 3.9. Biomaterials, Surfaces, and Biological Response

From a functional-biomaterials perspective, the surface and material evidence explains how the mini-implant behaves as a temporary device exposed to insertion trauma, mechanical loading, oral fluids, biofilm, and removal. These studies are not primarily site-selection studies; instead, they clarify why material, surface roughness, coatings, corrosion, ion release, and degradation may modify insertion stability, loaded stability, removal torque, and biological response.

In this section, “biomaterials” refers to the mini-implant material and surface characteristics, including titanium or stainless-steel alloys, roughness, coatings, corrosion, surface degradation, ion release, and tissue response. These studies do not usually determine the palatal insertion site directly, but they explain why two screws placed in similar bone may behave differently under insertion, loading, and removal. The mapped biomaterials evidence is summarized in Table 8.

Additional biomaterial and retrieved-device studies further supported this interpretation by evaluating new and recovered temporary anchorage devices, corrosion resistance, antimicrobial TiO_2_ nanotube surfaces, metallurgical and torsional properties, surface and elemental changes after clinical use, tissue integration under mechanical loading, recycled titanium mini-implants, finite-element stress fields, and the influence of implant material on primary stability [415,416,417,418,419,420,421,422,423,424].

### 3.10. Failure Modes, Safety and Reporting Gaps

Failure mechanisms mapped across the evidence base can be grouped into mechanical, biological, anatomical, digital/workflow, and reporting categories. A scoping review should not stop at reporting that failures occur; it should classify which part of the Bone–Screw–Force interface is most likely responsible and identify which variables future studies should report. The corresponding failure-mode framework is summarized in Table 9.

### 3.11. Integrated Clinical Decision Matrix

The decision framework should be read as a synthesis of the evidence rather than as a rigid clinical protocol. It assigns a first-line site based on the interaction between anatomical risk, load magnitude, vector control, and screw strategy. The most important feature of the framework is that it separates low/moderate orthodontic anchorage from high-load orthopedic expansion. The integrated decision matrix is presented in Table 10.

### 3.12. Synthesis of Evidence Gaps and Proposed Reporting Minimum

The most important gap is not absence of studies but lack of comparability. Many studies report site, screw, or appliance variables incompletely. Palatal studies frequently define regions differently; mechanistic studies often use non-palatal bone or synthetic models; MARPE studies vary in maturation criteria and appliance design; and digital planning studies report different deviation measures. These limitations do not invalidate the evidence, but they prevent simple thresholds from being generalized.

The proposed minimum reporting set is provided in Supplementary Table S4 and is summarized narratively in the Results and Discussion.

### 3.13. Comparative Synthesis Across Evidence Families

Detailed study-level comparisons for direct palatal anatomy, MARPE, digital workflows, primary and loaded stability, and biomaterials are provided in Supplementary Tables S6–S10. Direct human palatal clinical and imaging studies informed site-specific conclusions, whereas non-palatal clinical, finite-element, animal, cadaveric, in vitro, and synthetic-bone studies were retained only for mechanistic interpretation or hypothesis generation. Heterogeneity in models, outcomes and reporting precluded pooling and the derivation of universal numerical thresholds. Numerical values were reported only when directly traceable to the full text, a table, a figure, or a verified online full-text source.

## 4. Discussion

### 4.1. Principal Interpretation

The central finding of this scoping review is that palatal mini-implant selection cannot be reduced to a site map. Site maps are necessary, but they are only the anatomical component of a wider interface. Palatal CBCT studies define the substrate, PPSAIS and posterior-palatal studies illustrate the limits of anatomy-only extrapolation, and screw-design or insertion-torque studies show that stability is modified by device geometry, intraosseous engagement, and insertion protocol [4,9,14,15,16,17,18,19,20]. This integrated interpretation also explains why MARPE requires a separate high-load logic and why digital workflows become more relevant when trajectory, appliance fit, and cortical engagement must be controlled simultaneously [21,22,23,24,25,26,29,30,31,32,33].

### 4.2. Site Choice Is Conditional Rather than Absolute

The anterior palate and anterior paramedian region remain the best-supported routine sites, but the evidence does not justify a universal one-site recommendation. T-zone and anterior-palatal guidelines support routine skeletal anchorage, whereas CBCT mapping demonstrates variation by region, age, sex, facial pattern, mucosal thickness, and canal/root proximity [2,3,4,5,7,8,9,10,11,12,13,36,37,38]. Median insertion, T-zone placement, third-ruga placement, posterior palate, and PPSAIS therefore solve different problems and create different risks; the clinician should first define the appliance objective and then select the site that provides adequate bone, manageable mucosa, safe trajectory, and favorable force delivery [9,10,11,12,38,51,52,53,54,55,56].

### 4.3. MARPE as a High-Load Stress Test

MARPE exposes the limits of site-only reasoning. The screw is not merely resisting orthodontic tooth movement; it participates in an orthopedic load path involving the expander, arms, rings, palatal bone, nasal floor, and circummaxillary sutures [21,22,23,24,25,26,69,71]. Clinical and finite-element studies on tooth-bone-borne versus bone-borne expansion, microimplant displacement, pterygomaxillary response, bicortical or tricortical engagement, and circummaxillary effects show that expansion success depends on patient maturation, device rigidity, and force transmission rather than screw position alone [69,71,112,113,130,131,132,133,134,135,136,137,138,140,141,142,143,144,145,146,147]. In mature patients, the load may exceed the tolerance of the screw–bone interface or appliance framework before the desired skeletal response occurs, which justifies separate MARPE reporting and a separate decision logic [117,132,133,134,135,136,137,138,140,141,151].

### 4.4. Digital Planning Should Be Risk-Proportional

Digital planning should not be promoted as mandatory for every simple anterior palatal screw, nor dismissed as unnecessary technology. Its value is proportional to anatomical risk, biomechanical demand, and the need for precise appliance fit. Digital MARPE planning, CAD/CAM or printed insertion guides, lateral-cephalogram/intraoral-scan approaches, and step-by-step 3D accuracy analyses indicate that guidance is most useful when planned and actual trajectories must be tightly controlled [29,30,31,32,33]. In low-risk anterior palatal sites, experienced freehand placement may be acceptable; in PPSAIS, adult MARPE, special anatomy, narrow or high palates, canal proximity, or interradicular corridors, volumetric planning and guided insertion become much more defensible [9,11,31,32,33,80,81].

### 4.5. Biomaterials Relevance

The biomaterials literature is important for this manuscript because it explains how temporary anchorage devices behave as loaded materials in living tissues. Surface roughness, coatings, nanotubes, photofunctionalization, material choice, corrosion, sterilization, retrieved-device changes, and biological markers all modify the screw–tissue interface [14,34,35]. At the same time, surface-treated or modified devices do not automatically translate into superior palatal clinical performance; the evidence is strongest for mechanisms such as removal behavior, bone contact, corrosion, surface degradation, microdamage, and biological response, and weaker for direct site-specific recommendations [34,35,309,366,368,369,370,371]. The correct use of this evidence is therefore mechanistic support and future direction, not direct proof for choosing one palatal insertion zone over another.

### 4.6. Limitations of the Evidence and of This Review

The evidence is heterogeneous in terminology, measurement method, model type, and outcome definition. Direct human palatal clinical and anatomical evidence was distinguished from indirect mechanistic evidence because imaging-only studies often lack survival outcomes, experimental studies frequently use synthetic or non-palatal substrates, and many clinical reports omit screw dimensions, torque, mucosal thickness, force magnitude, or consistent failure definitions [4,5,6,7,8,9,10,11,12,13,14,15,16,17,18,19,20,21,22,23,24,25,26,44,45,46,47,48,69,70,71]. These limitations precluded pooling and restrict the generalizability of numerical thresholds. Detailed evidence-family inventories are therefore provided in the Supplementary Materials, whereas the main text retains only decision-relevant synthesis. Prospective assessment of reproducibility and clinical utility is required before prescriptive use.

### 4.7. Practical Implications

For routine palatal anchorage, anterior paramedian/T-zone sites remain pragmatic first-line options, supported by anatomical guidelines, CBCT mapping, and clinical experience [2,3,4,5,7,12,38]. For posterior vertical control or PPSAIS-based mechanics, CBCT may be considered when clinical and conventional radiographic assessment cannot adequately determine bone availability, anatomical risk, or insertion trajectory [9,10,11,81,90]. For adult MARPE, site selection should incorporate skeletal maturation, cortical engagement, appliance rigidity, and risk of asymmetric or non-opening response [21,22,23,24,25,26,69,71,112,113,130,131,132,133,134,135,136,137,138,140,141,142,143,144,145,146,147]. For biomaterial and screw design choices, larger or more retentive designs should be balanced against fracture, root proximity, tissue irritation, and removal considerations [14,15,16,17,18,19,20,34,35,268,278,426].

## 5. Conclusions

Palatal mini-implant performance should be framed as a Bone–Screw–Force interface problem rather than as a site-only problem. Direct palatal evidence supports anterior and anterior paramedian regions for many routine applications, but posterior sites, PPSAIS, and MARPE screw locations may warrant patient-specific imaging and biomechanical planning when conventional assessment is insufficient. Mechanistic evidence shows that primary and loaded stability depend on cortical and trabecular substrate, mucosal thickness, effective intraosseous length, screw geometry, material, surface, insertion torque, pilot-hole strategy, loading direction, and biological response. Digital workflows are best understood as technologies for controlling trajectory and appliance fit.

## Figures and Tables

**Table 1:** PCC framework for the scoping review.

Component	Operational Definition	Role in Synthesis
Population/participants	Orthodontic patients receiving, or being evaluated for, palatal orthodontic mini-implant anchorage, together with human palatal anatomical or imaging datasets directly relevant to placement.	Defines the direct human clinical and anatomical evidence base.
Concept	Interactions among palatal bone and soft-tissue substrate, mini-implant design and insertion variables, and load-specific mechanics in relation to primary or loaded stability, survival, failure, and site selection.	Defines the mechanism-centred synthesis.
Context	Palatal skeletal anchorage for sagittal, vertical, and transverse orthodontic or dentofacial-orthopaedic applications, including anterior or paramedian anchorage, posterior palate/PPSAIS, and MARPE.	Defines the site- and application-specific clinical context.

Animal, cadaveric, in vitro, synthetic-bone, and finite-element studies were not part of the direct PCC population. They were retained in a separate supporting stream solely for mechanistic interpretation and hypothesis generation.

**Table 2:** Direct palatal anatomical evidence: site, method, and synthesis role.

Evidence Family	Design/Method	Region or Site	Numeric or Comparative Signal	Role in Review	References
Anterior palatal CBCT maps	CBCT grids with multiple measurement points	Anterior palate, median and paramedian zones	Highest values generally anterior; one detailed map reported 180 patients and 88 palatal points, with maximum mean thickness at a defined anterior paramedian coordinate.	Provides the numeric basis for the schematic site map and site-specific matrix.	[4,5,9,10,51]
PPSAIS and posterior palate	CBCT posterior supra-alveolar assessment	Between the second premolar and first molar; posterior paramedian slopes; first- and second-molar regions	Posterior bone may be useful but is more variable; thick mucosa can reduce effective intraosseous engagement.	Supports patient-specific three-dimensional assessment rather than generic posterior placement when conventional assessment is insufficient.	[11,12,52,53,54]
Appliance-specific palatal anchorage sites	CBCT assessment of distalizer, mesializer, and expansion-framework insertion regions	Anterior paramedian, posterior palatal, and appliance-defined palatal positions	Bone, mucosa, and total tissue thickness differ by appliance design; sites for sliders, expanders, and posterior support should not be pooled.	Links anatomy to palatal appliance design and application-specific anchorage.	[13,36,55,56,57]
Nasopalatine canal and incisal region safety	CBCT canal morphology and distance mapping	Median/anterior palate near incisive canal	Wide anatomical variation means median insertion cannot be assumed risk-free.	Supports CBCT or careful 2D/3D planning when close to canal.	[37,39,58,59,60]
Patient-related anatomical modifiers	CBCT/CT subgroup and population-specific mapping	Anterior, median, paramedian, and posterior palate across age, maturation, facial-pattern, sex, and population groups	Bone thickness, density, palatal height or width, vault morphology, and safe-zone reliability vary with age, maturation, facial pattern, sex, population, and ethnicity.	Supports stratified interpretation and cautions against universal coordinate or screw-length thresholds.	[21,61,62,63,64,65,66,67,68,69,70,71,72,73,74,75,76,77,78,79,80,81,82,83,84]
Special anatomy	CBCT studies in cleft, syndromic, or high/narrow palates	Altered palatal vault, scarred tissues, or atypical bone distribution	Generic safe-zone maps are less reliable.	Consider CBCT when a defined three-dimensional diagnostic question cannot be resolved adequately by clinical and conventional radiographic assessment and when the result is expected to affect management.	[66,67,68,69,70]
Special anatomy, posterior palate, and supra-alveolar corridors	CBCT/CT studies focused on special anatomy, posterior access, PPSAIS, or safety landmarks	Posterior palate, palatal alveolar corridors, incisive canal/root proximity and special populations	Highlights areas where average anterior-palate rules do not apply and where patient-specific three-dimensional imaging may be warranted when conventional assessment is insufficient.	Supports posterior/PPSAIS caveats, safety rules, and supplementary site-specific mapping.	[22,23,40,85]

**Table 3:** Soft tissue and safety landmarks that modify effective screw engagement.

Variable	Why It Matters	Clinical Consequence	Evidence Synthesis
Mucosal thickness	Influences the transmucosal path, collar requirement, and available threaded bone engagement; the relationship is geometric rather than a simple subtraction.	Screw and collar selection should account for soft-tissue thickness together with threaded length, insertion angle, trajectory, and intended cortical engagement.	CBCT-based and ultrasonographic soft-tissue thickness studies should be charted separately from bone-depth studies, because mucosal thickness affects effective intraosseous screw engagement and transmucosal collar selection.
Incisive canal/nasopalatine bundle	Median anterior insertion may approach canal anatomy.	Median palatal screws require canal-aware planning when placed anteriorly.	Distance and morphology studies justify individualized planning.
Root proximity	Palatal alveolar/interradicular sites and anterior paramedian placement can approach roots depending on trajectory.	Freehand placement is less defensible in narrow or high-risk anatomy.	Root-contact studies should inform digital trajectory control.
Keratinized mucosa and hygiene	Soft-tissue inflammation can compromise survival even when bone is adequate.	Monitoring is required, especially for long-term appliances and thick mucosa.	Failure studies should report soft-tissue events explicitly.

**Table 4:** Site-specific palatal matrix for biomechanically driven mini-implant selection.

Category	Site/Subcategory	Substrate and Access	Typical Applications	Success/Evidence Signal and Planning Implication	References
A. Anterior palate/T-zone complex	A1. Median anterior palate	Central, root-free route; canal and suture anatomy must be considered.	Indirect anchorage, midline-supported appliances, sagittal mechanics and selected anterior-palatal frameworks.	Generally reliable for routine anchorage when canal/suture risk is respected; not automatically safer than paramedian placement.	[38,86,87,88,89,90,91,92,93,94]
A. Anterior palate/T-zone complex	A2. Anterior paramedian/third-ruga region	Clinically practical landmark with favorable access, keratinized mucosa and reduced root risk compared with interradicular sites.	Beneslider-type appliances, sliders, MARPE anterior screws, indirect anchorage, and selected canine traction.	Often preferred for routine anchorage when anatomy is favorable; success in anterior-palatal series is generally high but should be interpreted by screw system and follow-up.	[41,95,96,97,98]
A. Anterior palate/T-zone complex	A3. Median posterior palate	More posterior midline bone and mucosa; access and effective length may become less favorable.	Selected posterior anchorage or expansion-framework support when imaging confirms substrate.	Use selectively; success evidence is less mature than anterior/third-ruga data.	[90,91,92,93,94,99,100,101,102]
A. Anterior palate/T-zone complex	A4. Paramedian posterior palate	Posterior paramedian bone may support selected posterior vectors, but bone depth decreases and mucosa may thicken.	Posterior intrusion support, posterior anchorage, and selected posterior expansion support.	Consider patient-specific three-dimensional assessment when clinical and conventional radiographic evaluation is insufficient; this site is not interchangeable with anterior paramedian anchorage.	[24,99,100,101,102]
B. Footprint/posterior palatal zone	Footprint-type posterior palatal region	Posterior palatal zone used in some appliance concepts; definitions vary across studies.	Posterior support for selected sliders, distalization/intrusion mechanics and expansion frameworks.	Promising for posterior force vectors, but evidence is terminology-dependent and should be separated from PPSAIS and posterior paramedian data.	[103,104,105,106,107]
B. Posterior supra-alveolar corridor	PPSAIS	Palatal posterior supra-alveolar site that may provide posterior support when conventional posterior paramedian bone is limited.	Posterior force vectors, selected distalization/intrusion mechanics, and possible MARPE posterior support when patient-specific assessment confirms adequate substrate.	Evidence is less mature than anterior palate; use selectively and consider patient-specific three-dimensional imaging when conventional assessment is insufficient.	[103,104,105,106,107]
C. Palatal alveolar/interradicular zone	Palatal alveolar/interradicular corridors	Local tooth-adjacent corridors where root proximity becomes central.	Local tooth movement, adjunctive traction, selected distalization mechanics.	Use only with strict imaging and trajectory control; success depends strongly on root clearance.	[108]
D. MARPE screw zones	Anterior and posterior MARPE screw positions	Screw position is determined by appliance design, skeletal maturation, cortical engagement, and available bone.	High-load transverse expansion with multi-screw framework support.	Success/opening rates are heterogeneous and depend on age, suture maturation, activation protocol, screw number/position, and mono-/bicortical engagement.	[109,110,111,112,113]

**Table 5:** MARPE high-load evidence map.

Evidence Family	Study Designs	Variables Charted	Interpretation Limits	References
Conventional tooth-bone-borne MARPE	Clinical cohorts and CBCT outcome studies	Screw number, tooth support, skeletal/dental expansion, tipping, periodontal effects, and activation protocol.	Tooth support confounds interpretation of purely skeletal anchorage; activation rate should be reported as turns/day or mm/day.	[109,110,111,112,113]
Named Maxillary Skeletal Expander appliances	Clinical studies, CBCT follow-up, and finite-element analyses	Screw position, skeletal expansion, circummaxillary response, suture opening, displacement, and cortical engagement.	Outcomes depend on age, sutural maturation, activation protocol, and screw engagement; use the acronym only when the included study used it.	[130,131,132,133,134]
Bone-borne or hybrid expanders	Clinical, in vitro, and finite-element studies	Screw-only support, appliance rigidity, force transmission, stress distribution, and screw displacement.	Useful for isolating skeletal anchorage but less common clinically and highly design-dependent.	[130,131,132,133,134,135,136,137,138]
Bicortical/tricortical engagement strategies	Finite-element and clinical planning studies	Cortical engagement, stress distribution, displacement, stability, and nasal-floor relationship.	Mechanistic evidence often exceeds direct clinical validation.	[130,131,132,133,134,139]
Activation protocol and expansion rate	Clinical protocols and appliance-design studies	Turns per day, quarter-turns per day, millimeters of expansion, consolidation time, and activation rhythm.	Expansion activation should not be pooled with continuous orthodontic force in grams or newtons.	[109,110,111,112,113,135,136,137,138]
Complications and high-load failures	Case series, CBCT follow-up, and complication reports	Non-opening, asymmetry, fracture, craniofacial stress, screw displacement, and appliance deformation.	Supports risk stratification, monitoring, and conservative activation protocols.	[140,141,142,143,144]
Stability and post-expansion response	Follow-up, relapse, suture repair, and bone-healing studies	Repair and retention as biological phases of the high-load interface.	Evidence remains limited and should be reported separately from immediate opening.	[26,145,146,147,148]
Age and success predictors	Retrospective CBCT studies and predictor analyses	Midpalatal suture maturation, palatal bone thickness, success, and failure.	Comparability is limited when maturation staging, success criteria, and appliance protocols differ.	[149,150,151,152,153]

**Table 6:** Digital planning and guided insertion evidence map.

Digital Category	Study Types	Variables Extracted	Interpretation	References
Static CAD/CAM palatal guides	In vivo, ex vivo, cadaveric, and clinical guided-insertion studies	Coronal/apical deviation, angular deviation, guide support, and laboratory versus clinical transfer error.	Supports reproducible trajectory transfer and planned appliance fit; benefit depends on clinical risk.	[154,155,156,157,158,159,160,161,162,163]
One-visit appliance and transfer-cap workflows	Digital planning linked to transfer caps, prefabricated, or simultaneously delivered appliances	DICOM-STL matching, guide fabrication, passive fit, delivery timing, and screw-appliance compatibility.	Links planned screw position to appliance delivery and interface control.	[30,31,32,164,165,166,167,168,169,170]
Dynamic navigation and mixed-reality workflows	Navigation, augmented-reality, and mixed-reality accuracy studies	Angular and linear deviation, entry-point error, calibration, operator effect, and learning curve.	Potentially useful for higher-risk trajectories; availability and clinical validation remain limited.	[171,172,173,174,175,176,177,178,179,180]
Reduced-radiation planning	Lateral cephalogram plus intraoral scan or selected 2D/3D fusion workflows	Root clearance and anterior paramedian safety checks.	May be acceptable for selected low-risk anterior sites; consider CBCT in complex cases when a defined three-dimensional question cannot be resolved by conventional assessment.	[181,182,183,184,185]
AI-assisted mapping and safety prediction	Segmentation, site-identification, and prediction studies	Bone/mucosal mapping, candidate-site identification, prediction accuracy, and safety limits.	Future-oriented; clinical validation against actual insertion and outcomes remains limited.	[80,186,187,188,189,190,191,192,193]
Guide material, support, and sterilization effects	In vitro and clinical guide-material studies	Material stability, sterilization distortion, guide support, and socket length.	Links digital workflow accuracy to guide design and material performance.	[33,194,195,196,197]

**Table 7:** Determinants of primary and loaded stability.

Determinant Family	Representative Extraction Signal	Decision-Level Synthesis	Evidence Role	References
Cortical thickness and cancellous density	Mechanistic studies consistently linked higher cortical thickness/density to higher insertion torque or anchorage resistance; one synthesis range often cited for adequate cortical support is approximately 1.0–2.0 mm, but cancellous architecture also modified stability.	Bone quantity alone is insufficient; cortical shell, trabecular architecture, and local density should be interpreted together.	Core determinant of primary stability.	[14,15,16,247,248,249,250,251,252,253,254]
Effective intraosseous length	In one mechanistic series, 3 mm intrabony length was significantly less stable than longer engagement; thick palatal mucosa can reduce effective bony engagement despite adequate nominal screw length.	Report mucosal thickness and intraosseous thread engagement, not only nominal screw length.	Links soft tissue to screw selection.	[16,18,19,255,256,257]
Diameter, material, and fracture resistance	Palatal mini-implants and MARPE screws are generally wider/longer than many interradicular buccal miniscrews. Diameter should therefore be extracted separately for palatal, interradicular, and MARPE applications rather than averaged across all locations. Mechanical series show that larger diameter improves fracture resistance; one large study reported that diameter explained 90.3% of fracture-torque variation.	Increasing diameter may improve mechanical safety, but anatomical demand, mucosal thickness, and canal/root constraints limit indiscriminate enlargement.	Device-selection determinant.	[249,258,259,260,261,262,263,264,265,266,267]
Thread geometry, taper, and flutes	FEA and pull-out studies evaluated pitch/depth, taper, and flute geometry; thread shape can improve pull-out strength or stress distribution in models, but effects vary by substrate.	Thread design should be treated as a context-dependent modifier, not a universal solution.	Design-level determinant.	[268,269,270,271,272,273,274,275,276,277]
Pilot-hole and predrilling protocol	Pilot-hole and predrilling data are mostly derived from mechanistic, animal, or non-palatal/interradicular models. Ratios and drill diameters must therefore be interpreted as screw–bone mechanics rather than direct palatal prescriptions.	For palatal mini-implants, pilot-hole selection should be system-specific and should account for screw diameter, cortical thickness, bone density, and intended engagement pattern.	Insertion-protocol determinant; not a universal palatal rule.	[20,278,279,280,281,282,283,284,285]
Insertion angle and force direction	Oblique insertion may increase cortical contact in some contexts, but evidence from non-palatal and interradicular models cannot be transferred directly to palatal anchorage without considering site, screw length, cortical thickness, and force vector.	Insertion angle should be selected according to cortical thickness, anatomical risk, effective thread engagement, and intended loading vector.	Trajectory determinant.	[286,287,288,289,290]
Immediate/early loading and loaded stability	Animal and clinical studies show that insertion stability, loaded stability, removal torque, and remodeling are not interchangeable outcomes.	Primary stability should not be used as a proxy for long-term loaded performance without follow-up.	Time-dependent determinant.	[280,281,291,292,293,294,295]
Root/canal proximity and safety	Root contact length was associated with failure risk in safety studies; one report suggested the odds of failure approximately doubled per additional millimeter of root contact.	Trajectory control and imaging may dominate over raw bone thickness when roots or canals are approached.	Safety determinant.	[34,250,251,296,297,298,299,300,301,302,303]
Surface treatment and biological response	Surface-treated miniscrews showed higher removal torque or bone contact in some animal/clinical studies, but survival superiority was inconsistent; one split-mouth trial reported 90.3% vs. 83.9% survival without significant difference.	Surface engineering is promising but not yet sufficient for palatal site recommendations.	Biomaterial determinant.	[304,305,306,307,308,309,310,311,312,313,314,315,316,317,318,319,320,321,322,323,324]
Operator, measurement, and reporting factors	Learning curve, RFA/ISQ devices, torque meters, and mobility criteria vary across studies.	Future studies should separate biological failure, operator effects, and measurement method.	Reporting determinant.	[34,294,295,296,297,325,326,327,328,329]

Note: Data from interradicular or buccal miniscrew studies were used as supporting mechanistic evidence only. They should not be interpreted as direct dimensional recommendations for palatal mini-implants or MARPE screws.

**Table 8:** Biomaterials, surface modification, and biological response evidence.

Biomaterial or Surface Topic	What Was Tested	Main Outcome Mapped	Clinical Meaning for Palatal Mini-Implants	Caution and Key References
Titanium, stainless steel, and alternative alloys	Mechanical strength, bending, fracture torque, corrosion, surface alteration, and ion release.	Material affects mechanical safety, fracture behavior, and surface degradation after use.	Material choice matters most when combined with screw diameter, thread design, and cortical density.	Material alone does not determine clinical success [309,366,367,368,369,370,371,372,373,374,375,376].
Roughened, acid-etched, and sandblasted surfaces	Surface roughness, bone-to-implant contact, removal torque, survival, and surface integrity.	Rougher surfaces may increase bone contact or removal torque.	Surface modification may improve interface contact in selected contexts.	Higher removal torque is not always clinically desirable for temporary anchorage [310,311,312,313,314,370,371,377,378,379,380,381].
Anodization, nanotubes, and calcium-phosphate layers	TiO_2_ layers, nanotube arrays, calcium-phosphate coating, antimicrobial, or drug-loaded surfaces.	These strategies aim to improve osseointegration, antimicrobial behavior, or biological response.	Promising for future device optimization.	Most evidence is animal, in vitro, or non-palatal; clinical palatal benefit remains unproven [313,314,315,316,317,382,383,384,385,386].
Photofunctionalization and photobiomodulation	UV treatment, laser/photobiomodulation, and surface activation.	Some studies report improved contact, stability, or healing response.	May be an interface modifier in future temporary anchorage systems.	Evidence is heterogeneous and not yet sufficient for routine clinical recommendation [318,319,320,321,322].
Biofilm, inflammation, and biomarkers	Microbiome, peri-miniscrew inflammation, MMP-8, and tissue response.	Failure may be biological as well as mechanical.	Hygiene, soft-tissue control, and inflammation monitoring remain important.	These outcomes are not consistently reported in palatal studies [35,323,324,387,388,389,390,391,392,393,394].
Retrieved-device degradation and corrosion	Surface scratches, elemental changes, corrosion, deposits, and surface deterioration after clinical use.	Mini-implants can undergo surface alteration during insertion, loading, and removal.	Supports the concept of a temporary device exposed to mechanical and biological degradation.	Findings are descriptive and often system-specific [395,396,397,398,399,400,401,402,403,404,405,406,407,408,409].
Coatings with antimicrobial or bioactive intent	Silver, chitosan, hydroxyapatite, zinc oxide, and other coatings.	These coatings aim to reduce infection or improve bone response.	Interesting future direction for biomaterial design.	Clinical orthodontic evidence remains preliminary [410,411,412,413,414].

**Table 9:** Failure modes and reporting implications.

Failure Category	Likely Interface Mechanism	Minimum Data Required	Synthesis Use	References
Early mobility or loosening	Inadequate cortical engagement, poor bone quality, short effective intraosseous length, excessive early load.	Report insertion torque, screw dimensions, site, mucosa, load timing, and failure timing.	Table 7 and clinical extraction fields.	[16,256,272,291]
Root contact, canal encroachment, or unsafe anatomical proximity	Actual root contact, canal impingement, or an insertion trajectory too close to critical anatomy may increase mobility, biological irritation, or failure risk.	Report imaging method, planned and actual trajectory, root/canal clearance, and whether contact or encroachment occurred.	Safety and digital-planning outcome; proximity alone should be interpreted as risk rather than failure.	[34,250,296,297]
Soft-tissue inflammation/overgrowth	Thick mucosa, short collar, hygiene difficulty, appliance impingement.	Separate mucosal irritation from screw–bone instability.	Soft-tissue interface table.	[10,11,374]
Screw fracture or over-torque	Dense cortical bone, undersized pilot hole, small diameter, brittle design, or excessive torque.	Report maximum torque, fracture torque, material, and insertion protocol.	Biomaterials/failure table.	[249,251,270,368]
MARPE non-opening or asymmetric opening	Suture maturation, posterior resistance, screw mobility, appliance deformation, uneven force transfer.	Report maturation, activation, screw engagement, appliance design, and skeletal opening pattern.	MARPE high-load model.	[132,136,146,425]
Evidence/reporting heterogeneity	Non-standard site terminology, missing screw dimensions, incomplete load description, unclear follow-up.	Adopt a minimum reporting set: site, screw, insertion, load, imaging, failure definition, and follow-up.	Future research agenda.	[44,45,46,49]

**Table 10:** Author-derived Bone–Screw–Force conceptual synthesis matrix.

Clinical Objective	Load Level	Preferred Site Logic	Screw/Appliance Strategy	Imaging/Guidance Requirement	Main Risk
Routine indirect anchorage	Low to moderate	Anterior palate, T-zone, or anterior paramedian region when anatomy is favorable.	Single screw or paired screws; avoid unnecessary posterior placement.	Clinical and conventional radiographic assessment is generally sufficient; consider CBCT only if a defined three-dimensional question remains unresolved.	Soft-tissue irritation, poor effective engagement.
Molar distalization	Moderate	Anterior paramedian/T-zone for slider access and rigid connection.	Paired palatal screws or palatal plate/slider depending on force vector.	Consider CBCT when posterior bone, anatomical risk, or the planned trajectory cannot be assessed adequately by conventional methods.	Tipping, rotation, anchorage-unit deformation.
Molar mesialization	Moderate	Anterior paramedian or T-zone with rigid connector.	Coupled screws if large sagittal force or moment control required.	Consider CBCT when posterior bone, anatomical risk, or the planned trajectory cannot be assessed adequately by conventional methods.	Vertical side effects and rotational moments.
Posterior intrusion/open bite	Moderate to high; commonly about 200–400 g depending on tooth/unit, anchorage design, and periodontal limits.	Posterior palate or PPSAIS may be biomechanically attractive; anterior sites may require longer lever arms.	Consider buccal-palatal control to reduce tipping.	Consider CBCT when conventional assessment cannot adequately define posterior bone availability, anatomical risk, or insertion trajectory.	Palatal-only tipping, soft-tissue overgrowth.
Impacted canine traction	Low to moderate but prolonged	Anterior palate/paramedian route when it improves vector control.	Indirect anchorage and traction line planned around roots.	Consider CBCT when conventional imaging cannot adequately define impacted-tooth/root relationships or the planned trajectory.	Root contact, unfavorable traction vector.
MARPE adolescent	High orthopedic expansion; report activation rate as turns/day or mm/day. If adjunctive orthopedic traction is used, report force separately and do not pool it with expander activation.	Anterior/posterior paramedian screw positions according to expander design.	Multi-screw appliance; monitor opening, soft tissue, and screw mobility; activation commonly follows protocol-specific quarter-turn schedules.	Consider CBCT when patient-specific three-dimensional assessment of bone availability, cortical engagement, anatomical risk, or insertion trajectory is expected to alter planning.	Asymmetric opening, appliance deformation, screw mobility.
MARPE adult	High orthopedic expansion; do not assume one universal force level. Report activation protocol, resistance, maturation, and achieved opening.	Patient-specific multi-screw paramedian engagement; consider posterior support according to anatomy and appliance design.	Bicortical or enhanced engagement depending on anatomy and design; consider posterior support when indicated.	Consider CBCT when patient-specific three-dimensional assessment of bone availability, cortical engagement, anatomical risk, or insertion trajectory is expected to alter planning.	Non-opening, skeletal resistance, fracture, failure of appliance or screw.
Special anatomy	Variable	No generic site map should be assumed.	Individualized screw number, length, collar, and guide strategy.	Consider CBCT when conventional assessment cannot define the relevant three-dimensional anatomy and the result is expected to alter management.	Anatomical injury, insufficient bone, tissue problems.

Clinical recommendations are supported by direct human palatal evidence; indirect evidence informs mechanisms only.

## Data Availability

No new experimental or patient-level data were generated in this study. The evidence-mapping data supporting the synthesis are derived from published studies cited in the reference list and are summarized in the article, Supplementary File S1, and Supplementary Data S2.

## Supplementary Materials

The following supporting information can be downloaded at: https://www.mdpi.com/article/10.3390/jfb17080360/s1, Table S1: Eligibility logic and full-text decision rules; Table S2: Evidence streams and synthesis function; Table S3: Clinical force applications and Bone–Screw–Force interpretation; Table S4: Minimum reporting set for future palatal mini-implant studies; Table S5: Detailed primary-stability evidence families retained for transparency; Table S6: Key direct palatal anatomy studies and their contribution to site-specific interpretation; Table S7: MARPE high-load evidence and its distinction from routine palatal anchorage; Table S8: Digital workflow studies and extraction fields; Table S9: Primary and loaded stability evidence families and comparison fields; Table S10: Biomaterials and surface evidence: translational readiness for palatal mini-implant decision-making; Table S11: Complete database-specific search strategies; Table S12: PRISMA-ScR checklist; Supplementary Data S2: Record-level evidence-map inventory.

## Abbreviations

The following abbreviations are used in this manuscript:

BSF	Bone–Screw–Force
TAD	Temporary anchorage device
OMI	Orthodontic mini-implant
CBCT	Cone-beam computed tomography
CT	Computed tomography
PRISMA-ScR	Preferred Reporting Items for Systematic Reviews and Meta-Analyses extension for Scoping Reviews
PCC	Population/Participants–Concept–Context
MARPE	Miniscrew-assisted rapid palatal expansion
MSE	Maxillary Skeletal Expander
RPE	Rapid palatal expansion
SARPE	Surgically assisted rapid palatal expansion
PPSAIS	Palatal posterior supra-alveolar insertion site
FEA	Finite-element analysis
CAD/CAM	Computer-aided design/computer-aided manufacturing
STL	Stereolithography file format
DICOM	Digital Imaging and Communications in Medicine
IOS	Intraoral scan/intraoral scanner
RFA	Resonance frequency analysis
ISQ	Implant stability quotient
BIC	Bone-to-implant contact
MAO	Micro-arc oxidation
RBM	Resorbable blasting media
UV	Ultraviolet
TiO_2_	Titanium dioxide
HA	Hydroxyapatite
MMP-8	Matrix metalloproteinase-8
