# Supplementary material for: Bone–Screw–Force Interactions in Palatal Orthodontic Mini-Implants: A Scoping Review and Decision Framework for Primary Stability and Biomechanically Driven Site Selection
Source: J Funct Biomater. 2026 Jul 26;17(8):360. doi: 10.3390/jfb17080360 (PMC13513910; doi:10.3390/jfb17080360)
Supplement: Supplementary file 1 [file jfb-17-00360-s001.zip › jfb-4450877-supplementary.pdf]

Supplementary File S1.

Supplementary Table S1. Eligibility logic and full-text decision rules.

Decision level	Inclusion logic	Exclusion logic	Purpose
**Title/abstract**	Retain if the record addresses palatal anatomy, palatal mini-implants, MARPE/MSE, digital planning, primary stability, screw design, torque, surface, loading or failure.	Exclude if clearly unrelated, conventional implant-only, buccal-only without mechanistic relevance, opinion-only or conference abstract without usable information.	High-sensitivity screening.
**Full text**	Include if extractable Bone, Screw, Force, Digital, Biomaterial or Failure information is available.	Exclude if no extractable variable, no relevance to orthodontic mini-implants or no relation to palatal/direct or mechanistic evidence.	Eligibility and evidence charting.
**Numeric extraction**	Lock only values verified from full text/PDF/HTML with study locator where available.	Do not promote abstract-only values into final numeric tables unless full text cannot be retrieved and the value is explicitly marked.	Avoid artificial numeric certainty.

Supplementary Table S2. Evidence streams and synthesis function.

Evidence stream	Records processed	Main evidence types	Synthesis function
**P1 direct palatal evidence**	596	CBCT palatal anatomy, palatal mini-implant survival, MARPE/MSE, digital palatal guides, palatal appliances.	Site matrix, clinical decision matrix, MARPE/MSE model and palatal-specific statements.
**P2 supporting mechanistic evidence**	688	Torque, screw design, surface/material, pilot-hole, microdamage, FEA, in vitro/animal models, removal torque.	Primary stability mechanisms, biomaterials section, failure modes and future research.
**Numerical charting**	All retained evidence sources with verifiable quantitative outcomes relevant to a prespecified Bone–Screw–Force variable.	Values were used only when traceable to the full text, table, figure or verified online full-text source.	No fixed 120-record sampling stratum was used.

Supplementary Table S3. Clinical force applications and Bone-Screw-Force interpretation.

Application	Typical systems	Variables to chart	Decision implication	References
Molar distalization, including clear-aligner and hybrid mechanics	Palatal sliders, distalizers, plates and clear-aligner adjuncts	Sagittal force vector, molar tipping and rotation, anchorage stiffness, appliance fit and vertical control.	Anterior paramedian/T-zone anchorage is often convenient when sagittal mechanics dominate; the force line and rotational control remain appliance-specific.	[27, 28, 42, 43, 102, 103, 106–108, 198, 200, 201, 203–207, 210, 211, 215, 225, 226]

Application	Typical systems	Variables to chart	Decision implication	References
Molar mesialization, space closure and reciprocal-force control	Mesialization systems, rigid palatal frameworks and indirect anchorage	Moment control, vertical side effects, anchorage coupling and appliance stiffness.	Paired or framework-based anchorage may reduce unwanted rotation when the force system generates substantial moments.	[104, 217, 218]
Posterior intrusion and open-bite correction	Palatal, buccal or dual anchorage configurations; intrusion transpalatal arches and clear-aligner adjuncts	Intrusive vector, buccopalatal tipping, root response, periodontal response and occlusal-plane control.	Palatal-only systems may require buccal balancing or other vector-control measures depending on the case.	[199, 202, 208, 209, 212–214, 219, 220, 223, 224]
Impacted tooth traction and indirect anchorage	Direct or indirect miniscrew anchorage for palatally displaced canine traction	Vector progression, tooth-movement speed, anchorage stability, root safety and appliance rigidity.	Palatal anchorage can support controlled traction when the vector and adjacent-root relationships are explicitly planned.	[105]
Class III protraction and high-load orthopedic adjuncts	Maxillary skeletal expanders, facemask combinations and hybrid orthopedic protocols	Load magnitude, anchorage distribution, skeletal response, sutural resistance and device deformation.	These high-load protocols should not be treated as interchangeable with routine palatal mini-implant anchorage.	[115, 233, 245]

Supplementary Table S4. Minimum reporting set recommended for future palatal mini-implant studies.

Domain	Minimum variables to report	Reason
**Patient/anatomy**	Age, sex, skeletal pattern, maturation, palatal depth/width, special anatomy.	Allows stratification of bone and force response.
**Site**	Named region, coordinate/reference system, median/paramedian distance, AP landmark, angulation.	Prevents non-comparable site terminology.
**Bone/soft tissue**	Total bone depth, cortical thickness, density if available, mucosal thickness.	Separates nominal screw length from effective intraosseous engagement.
**Screw**	Brand/system, material, diameter, length, thread/taper/collar, surface/coating.	Allows screw-design interpretation.
**Insertion**	Freehand/guide, pilot-hole, insertion angle, torque, mono-/bicortical engagement.	Allows stability mechanism interpretation.
**Force/appliance**	Force magnitude/direction, timing, appliance rigidity, loading duration.	Connects site to biomechanical demand.
**Outcomes**	Primary stability, mobility, survival, failure timing, complications, removal torque, follow-up.	Distinguishes early mechanical failure from loaded/biological failure.

Supplementary Table S5. Detailed primary-stability evidence families retained for transparency.

Determinant	Evidence types	Variables charted	Critical interpretation	References
**Cortical thickness and trabecular density studies**	Cadaveric, CBCT, in vitro and FEA studies	Cortical thickness, trabecular density, BMC/BMD, RFA/ISQ, insertion torque and pull-out strength.	Bone quantity matters, but cancellous architecture and density modify the effect of the cortical shell.	[15–18, 255, 256, 259, 260, 272, 276, 280, 291, 340]

Determinant	Evidence types	Variables charted	Critical interpretation	References
**Insertion torque and torque-window studies**	Clinical, animal and in vitro torque studies	Peak insertion torque, removal torque, fracture torque, mobility and success/failure timing.	Torque should be interpreted as an interface signal; excessive torque may increase microdamage or fracture risk.	[14, 19, 20, 256, 274, 278, 279, 287, 297, 331, 334]
**Pilot-hole, predrilling and self-drilling protocols**	Experimental, animal and clinical insertion-protocol studies	Pilot-hole diameter, predrilling depth, self-drilling/self-tapping mode, heat and microdamage.	Site preparation can improve insertion control but may reduce stability if overprepared.	[20, 251, 258, 261, 262, 283, 288, 290, 293, 295, 367, 426]
**Insertion angle and effective cortical engagement**	FEA, in vitro and clinical placement-angle studies	Angulation, cortical contact length, insertion depth and load direction.	Oblique insertion is not universally superior; benefit depends on bone thickness and force vector.	[12, 248, 251, 292, 294, 344, 349, 423]
**Thread design, pitch, depth and taper geometry**	FEA, mechanical and in vitro design studies	Thread pitch/depth, taper length, thread shape, flute geometry and pull-out resistance.	Geometry modifies stress distribution and anchorage resistance, but clinical behavior remains interface-dependent.	[17, 19, 247, 257, 264, 265, 268, 269, 275, 281, 330, 353, 354, 356, 363, 365]
**Screw diameter, length and effective intraosseous engagement**	Mechanical, animal and clinical studies	Diameter, nominal length, intrabony length, fracture resistance and removal torque.	Effective bone engagement is more informative than nominal length when mucosa is thick.	[16, 249, 270, 271, 278, 289, 335, 346, 358, 360, 364, 405, 407]
**Material choice and mechanical fracture resistance**	Mechanical testing and comparative material studies	Titanium, stainless steel, alternative alloys, bending, torsional and fracture torque.	Material interacts with diameter and dense bone; stronger devices may still carry anatomical risks.	[249, 271, 273, 289, 322, 346, 358, 364, 381, 386, 405, 407, 413, 418, 424]
**Pull-out strength and displacement under orthodontic load**	Mechanical tests, FEA and loaded-stability models	Pull-out force, displacement, drift, viscoelastic response and anchorage resistance.	Loaded stability may diverge from insertion stability when force direction or appliance stiffness changes.	[257, 259, 292, 294, 297, 330, 340, 356, 365]
**RFA/ISQ, mobility and longitudinal stability measures**	Clinical, cadaveric and animal studies	RFA, ISQ, damping capacity, mobility, removal torque and stability over time.	Different stability measures are not interchangeable and should be reported separately.	[266, 267, 277, 337, 340, 343, 388, 408, 409]
**Root proximity and anatomical safety studies**	CBCT, animal and clinical failure studies	Root contact length, canal/root distance, insertion trajectory and failure rate.	Anatomical safety can dominate design factors when roots or canals are approached.	[31, 96, 303, 325–328, 343, 344, 372, 389]
Microdamage and insertion-related biological trauma studies	Micro-CT, histology, OCT and thermal studies	Microcracks, bone voids, cortical compression and healing response.	Mechanical stability must be balanced against iatrogenic bone trauma.	[18, 296, 350, 396, 398, 399]
**Surface treatment, bone contact and removal behavior**	Animal, in vitro and retrieved-device studies	Surface roughness, BIC, removal torque, photofunctionalization and coatings.	Surface modification may alter integration and removal behavior, but clinical maturity is variable.	[34, 250, 252, 263, 284, 309, 318–324, 332, 333, 338, 339, 366, 368, 369, 373, 387, 397, 402]
**Reinsertion, reuse and sterilization effects**	Experimental and clinical studies of reused or reinserted miniscrews	Surface alteration, sterilization effect, reinsertion stability and torque changes.	Temporary devices may behave differently after reuse, reinsertion or surface damage.	[282, 286, 301, 355, 392, 410, 415, 421, 422]

Determinant	Evidence types	Variables charted	Critical interpretation	References
**Animal/cadaver validation of screw-bone contact**	Animal, cadaveric and histomorphometric studies	Bone-miniscrew contact, healing sequence, immediate loading and osseointegration markers.	Useful for mechanism, but not direct proof of palatal clinical outcome.	[255, 262, 263, 287, 319–322, 333, 367, 376, 388, 393, 397, 400, 420]
**Finite-element prediction of stress, strain and failure**	Patient-specific and generic FEA models	Stress, strain, screw position, force direction, cortical thickness and failure prediction.	FEA identifies mechanisms and hypotheses; clinical validation remains necessary.	[17, 247, 248, 292, 304, 306, 330, 372, 423]
**Clinical survival and failure-factor cohorts**	Retrospective and prospective clinical cohorts	Success/failure, age, site, arch, inflammation, operator factors and load timing.	Clinical cohorts anchor the mechanistic findings but often underreport device and force variables.	[34, 70, 102–108, 192, 301–303, 305, 307, 309, 318, 325, 328, 344, 345, 359, 362, 366, 386, 389–391, 397, 406]

Supplementary Table S6. Key direct palatal anatomy studies and their contribution to site-specific interpretation.

Study / cluster	Sample or method	Region	Extractable signal	Interpretive value	Refs
**Chhatwani et al. (2019)**	180 CBCTs; 88 palatal measurement points	Median and paramedian palate	n=180 CBCTs and 88 measurement points; maximum mean thickness was reported at an anterior paramedian coordinate (9.7 +/- 2.8 mm at 4 mm posterior to the incisive foramen in the cited map).	Numerical support for anterior/T-zone first-line planning; the value is a representative coordinate, not a universal threshold.	[4]
**Nucera et al. (2022; two studies)**	Posterior palatal CBCT mapping; multiple angulations	PPSAIS / posterior supra-alveolar region	The PPSAIS was assessed at multiple angulations (45, 60 and 75 degrees); mucosa was reported as thick and variable, often in the 4-7 mm range in posterior regions.	Consider patient-specific three-dimensional assessment for posterior supra-alveolar placement when conventional assessment cannot adequately define bone depth, mucosal offset or trajectory.	[9, 51]
Gibas-Stanek et al. (2023)	CBCT assessment of distalizer-related palatal sites	TopJet / Benefit-related regions	For one distalizer-related site (M4), total tissue thickness was 13.26 +/- 3.81 mm, bone thickness 8.01 +/- 3.53 mm and mucosa 5.25 +/- 1.85 mm.	Shows why total tissue thickness must be decomposed into bone and mucosa when selecting screw length/collar.	[5]
**Burak and Hilmi (2024)**	CBCT mucosal-thickness mapping	Anterior, middle and posterior palatal mucosa	n=60 CBCTs in one mucosal-thickness map; mucosa generally increased from anterior to posterior regions.	Soft tissue can reduce effective intraosseous length and should modify nominal screw-length recommendations.	[10]
**Schubert et al. (2024)**	CBCT safe-zone mapping	Paramedian/anterior palate	Safe-zone mapping incorporated bone volume, age, sex and insertion angle rather than a single coordinate.	Supports individualized planning and discourages one-size-fits-all coordinates.	[12]

Study / cluster	Sample or method	Region	Extractable signal	Interpretive value	Refs
**Zhao et al. (2011); Chen et al. (2021); Yu et al. (2021); Negrisoli et al. (2022); Nie et al. (2023)**	CBCT palatal thickness / MARPE positioning studies	MARPE anterior and posterior screw sites	MARPE anatomy studies repeatedly separated anterior and posterior screw positions and distances from the midpalatal suture; exact values should be extracted per appliance design.	Expansion screws require separate site analysis from routine anchorage.	[55, 56, 67, 68, 71]
Special-anatomy and morphology studies	CBCT studies of cleidocranial dysplasia, mouth breathing with a high-narrow palate, unilateral cleft lip/palate and facial-pattern or palatal-vault variation	Altered palatal vault or bone distribution	Direct comparisons document anatomy-specific differences in palatal support tissues; measurements should be interpreted within the relevant population and morphology.	Generic maps should not be transferred uncritically to non-standard anatomy; three-dimensional imaging should be used when the clinical question cannot be resolved by conventional assessment.	[39–41, 59, 62, 66, 67, 72, 74–76, 98]

Supplementary Table S7. MARPE high-load evidence and its distinction from routine palatal anchorage.

Cluster	Evidence type	Variables	Main synthesis	Minimum reporting requirement	Refs
**Adolescent MARPE**	Clinical CBCT cohorts and comparative studies	Suture opening, skeletal/dentoalveolar effects, periodontal response	Clinical comparative evidence includes high suture-opening proportions in growing or less mature patients; one RCT-level comparison reported midpalatal suture separation in 90% of RPE and 95% of MARPE cases.	Report maturation stage, age, screw dimensions, screw position, activation and consolidation.	[25, 132–135]
**Adult MARPE**	Clinical cohorts, case-control studies and high-load appliance studies	Success/failure, non-opening, asymmetric expansion, circummaxillary effects	Adult cohorts show that success depends on skeletal maturity, sutural resistance and appliance design; adult MARPE should be treated as high-load skeletal anchorage rather than routine palatal anchorage.	Report age, maturation/suture status, bicortical engagement, failure definition and need for adjunctive surgery.	[136–138, 140, 141]
**FEA and in vitro MARPE**	Finite-element models and mechanical testing of expanders	Stress distribution, displacement, arm height, mono-/bicortical engagement	FEA and bench studies compare mono-/bicortical or tricortical engagement, arm height and expander architecture; models show that force transmission changes with appliance design before clinical failure is visible.	Report material properties, boundary conditions, screw length/diameter, engagement and loading protocol.	[26, 146–149]

Cluster	Evidence type	Variables	Main synthesis	Minimum reporting requirement	Refs
**Custom/digital MARPE**	Digital/custom appliance studies	Fit, screw trajectory, guide or appliance design, skeletal response	Custom/digital MARPE studies include cohorts such as 42 patients aged 16-35 years in comparative custom-vs-conventional expansion work and digitally manufactured devices with planned screw trajectories.	Report planned versus achieved screw positions and appliance deformation, not only expansion amount.	[139, 150–153]
**Complications and biological response**	Case series and follow-up studies	Fracture, asymmetric opening, bone healing, suture repair	Follow-up studies include adult suture-repair evaluation around 16 months and reports of palatal/circummaxillary fracture or asymmetric expansion.	Classify failure as screw, appliance, suture/circum maxillary or biological.	[114–117, 425]

Supplementary Table S8. Digital workflow studies and extraction fields.

Digital family	What it measures	Core variables	Clinical interpretation	Caveat	Refs
**Static CAD/CAM palatal guides**	Planned vs achieved mini-implant position; examples include 52 palatal screws in 20 patients and 64 bicortical palatal screws in 33 subjects.	Linear/angular deviations; coronal/apical displacement; appliance/transfer-cap fit; one workflow reported laboratory angular deviation 2.12 +/- 1.62 degrees, clinical deviation 6.23 +/- 3.75 degrees and total deviation 5.70 +/- 3.42 degrees.	Useful for anterior palate, paramedian placement and one-visit appliance workflows.	Does not eliminate biological or force-related failure.	[30–32, 164, 165]
**Laboratory vs clinical error studies**	Separation of laboratory production error from clinical insertion error.	Angular deviation, guide seating, coronal/apical displacement; clinical execution can exceed laboratory error.	Important because the largest error may occur clinically, not digitally.	Requires clear reporting of planned and final position.	[171–175]
**Dynamic navigation / mixed reality**	Navigation accuracy and operator effect; pilot studies report angular/linear deviation and learning-curve variables.	Operator generation, morphology and calibration should be reported alongside deviation.	Promising for complex anatomy; not yet routine for every case.	Should report calibration, operator experience and morphology.	[181–185]

Digital family	What it measures	Core variables	Clinical interpretation	Caveat	Refs
**Reduced-radiation workflows**	Lateral ceph + IOS or virtual-plaster planning; one reduced-radiation protocol tested 20 patients with virtual 1.7 x 8 mm anterior paramedian screws.	Root safety and anterior paramedian trajectory; consider CBCT outside low-risk anterior regions when a defined three-dimensional question remains unresolved.	Potentially acceptable only in selected low-risk sites.	Consider CBCT for posterior/PPS AIS/MARPE/special anatomy when conventional assessment cannot resolve the relevant three-dimensional question.	[186–190]
**AI and automated mapping**	Segmentation and candidate-site algorithms; early AI studies focus on bone/mucosa mapping rather than clinical outcomes.	Report segmentation accuracy and planned site adequacy, but validate against actual insertion and survival.	Future tool for screening; clinical validation remains limited.	Should be validated against outcomes, not only segmentation accuracy.	[159–163]

Supplementary Table S9. Primary and loaded stability evidence families and comparison fields.

Mechanistic domain	Evidence type	Variables	Synthesis	Refs
**Cortical thickness / density**	Cadaveric, CBCT and in vitro studies	Insertion torque, BMC, cortical thickness, RFA/ISQ, pull-out force	Cortical thickness is central, but cancellous structure and density modify stability; adequate cortical support is often discussed around 1.0-2.0 mm, with CBCT-based measures predicting insertion variables in some models.	[15, 17, 255, 256, 259, 260, 272, 276, 280, 291, 340]
**Effective intrabony length**	Mechanical and finite-element studies	Intraosseous length, mucosal offset, cortical density, stability	Nominal screw length is less meaningful than effective thread engagement; 3 mm intrabony length was significantly less stable than longer engagement in one mechanistic study.	[16, 255, 256]
**Diameter and fracture resistance**	Mechanical tests and material comparisons	Fracture torque, bending, self-drilling performance	Larger diameter may reduce fracture risk but increases anatomical demand; in one large mechanical comparison, diameter explained 90.3% of fracture-torque variation.	[249, 270, 271, 289, 335, 346, 358, 364, 381, 407, 413]
**Thread shape and taper**	FEA and mechanical pull-out tests	Thread pitch/depth, taper, flutes, pull-out strength, stress	Thread pitch/depth and taper modify stress and pull-out behavior; reported optimal designs are model-dependent and should not be generalized without substrate details.	[247, 257, 264, 265, 268, 269, 275, 281, 330, 353, 354, 356, 363, 365]
**Insertion protocol**	Pilot-hole, self-drilling/tapping and thermal studies	Pilot diameter, insertion torque, microdamage, heat, stability over time	Pilot-hole strategy should be matched to bone density; ratios around 69-77% of external diameter and a 1.0 mm pilot hole for a 1.3 mm screw were reported as representative mechanical targets in selected models.	[20, 251, 258, 261, 262, 283, 288, 290, 293, 295, 367, 426]
**Loading timing and direction**	Animal, clinical and FEA studies	Immediate/delayed loading, direction, displacement, removal torque	Immediate/delayed loading, force direction and appliance stiffness alter loaded stability; primary stability, RFA/ISQ, removal torque and clinical survival should be reported separately.	[247, 297–299, 329, 345, 372, 383, 394, 420]

Mechanistic domain	Evidence type	Variables	Synthesis	Refs
**Root proximity and anatomical safety**	CBCT and animal/clinical studies	Root contact, distance, failure risk, healing response	Root proximity can dominate design factors; one safety study reported approximately doubled failure odds per additional millimeter of root contact.	[31, 96, 303, 325–328, 343, 344, 372, 389]

Supplementary Table S10. Biomaterials and surface evidence: translational readiness for palatal mini-implant decision-making.

Theme	Evidence type	Measured outcomes	Interpretation	Translational status	Refs
**Titanium vs stainless steel and alternative materials**	Mechanical and biological comparisons	Fracture torque, bending, torsional strength, surface integrity and histology; one 504-screw comparison found diameter explained most fracture-torque variation, while alloy explained a smaller fraction.	Material is clinically relevant for dense bone and fracture risk, but geometry and diameter often dominate mechanical behavior.	Moderate; often non-palatal.	[249, 273, 289, 313, 322, 381, 386, 404, 405, 413, 418, 424]
**Surface roughness / acid-etched / sandblasted**	Clinical, animal and in vitro studies	Removal torque, survival and bone-to-implant contact; a split-mouth trial reported 90.3% survival for sandblasted/acid-etched miniscrews versus 83.9% for controls, without significant survival superiority.	Surface roughness may increase interface engagement or removal torque, but clinical superiority is inconsistent.	Moderate; interpret cautiously.	[34, 252, 263, 284, 309, 318, 319, 332, 333, 338, 339, 366, 368, 369, 387, 397, 402]
**Nanotubes, calcium phosphate, hydroxyapatite, coatings**	Animal and in vitro biomaterial studies	Osseointegration, antimicrobial effect, surface chemistry and drug release; most evidence remains animal/in vitro.	Promising for future device design but not yet mature enough for site-specific palatal recommendations.	Low-to-moderate translational readiness.	[310–312, 315–317, 321, 371, 376, 379, 380, 384, 393, 400–403, 417]
Photofunctionalization and light-based surface activation	Surface activation, animal and clinical studies	Stability, bone contact, removal torque and healing; protocols vary across UV photofunctionalization and photobiomodulation studies.	May modify biological response, but clinical protocols remain heterogeneous.	Emerging.	[320, 323, 368, 397]
**Retrieved mini-implants / degradation**	Surface and elemental analysis after use	SEM/topography, corrosion, ion release, surface alteration and elemental changes in retrieved devices.	Highly relevant to JFB and device safety, especially for removal-stage biology and reuse/sterilization questions.	High relevance to JFB, but not site-specific.	[35, 313, 314, 324, 370, 374, 375, 378, 382, 410, 411, 415, 416, 419, 421, 422]

Theme	Evidence type	Measured outcomes	Interpretation	Translational status	Refs
**Biofilm, biomarkers and inflammation**	Clinical biological-response studies	Peri-miniscrew microbiology, MMP-8, soft-tissue inflammation and hygiene-related outcomes.	Explains non-mechanical failure pathways and should be integrated with mechanical stability data.	Requires standardized outcome definitions.	[298–300, 316, 329, 373, 377, 379, 384, 385, 403, 412, 417]

Supplementary Table S11. Complete database-specific search strategies.

Search date: 26 May 2026. Limits: no date, language or document-type restrictions.

Search-yield summary

Database	Block A	Block B	Block C	Block D	Block E	Total
PubMed/MEDLINE	393	305	905	106	313	2,022
Scopus	520	388	1,610	107	73	2,698
Web of Science Core Collection	507	489	1,269	145	386	2,796
**Total**	1,420	1,182	3,784	358	772	7,516

PubMed/MEDLINE

Block A — Palatal anatomical substrate (yield: 393)

(miniscrew*[tiab] OR "mini-screw"[tiab] OR "mini-screws"[tiab] OR miniimplant*[tiab] OR "mini-implant"[tiab] OR "mini-implants"[tiab] OR "mini implant"[tiab] OR "mini implants"[tiab] OR microimplant*[tiab] OR "micro-implant"[tiab] OR "micro-implants"[tiab] OR "micro implant"[tiab] OR "micro implants"[tiab] OR "temporary anchorage device"[tiab] OR "temporary anchorage devices"[tiab] OR TAD[tiab] OR TADs[tiab] OR "skeletal anchorage"[tiab] OR "Orthodontic Anchorage Procedures"[Mesh])
AND
(palate[tiab] OR palatal[tiab] OR "anterior palate"[tiab] OR midpalatal[tiab] OR "mid-palatal"[tiab] OR "median palate"[tiab] OR "paramedian palate"[tiab] OR "T-zone"[tiab] OR "T zone"[tiab] OR "posterior palate"[tiab] OR "palatal slope"[tiab] OR "palatal vault"[tiab] OR PPSAIS[tiab] OR "palatal posterior supra-alveolar"[tiab] OR parapalatal[tiab] OR "para-palatal"[tiab])
AND
("bone thickness"[tiab] OR "cortical thickness"[tiab] OR "cortical bone"[tiab] OR "bone density"[tiab] OR "bone depth"[tiab] OR "bone volume"[tiab] OR "mucosal thickness"[tiab] OR "soft tissue thickness"[tiab] OR CBCT[tiab] OR "cone beam computed tomography"[tiab] OR "computed tomography"[tiab])

Block B — Primary stability, survival and failure (yield: 305)

(miniscrew*[tiab] OR "mini-screw"[tiab] OR "mini-screws"[tiab] OR miniimplant*[tiab] OR "mini-implant"[tiab] OR "mini-implants"[tiab] OR "mini implant"[tiab] OR "mini implants"[tiab] OR microimplant*[tiab] OR "micro-implant"[tiab] OR "micro-implants"[tiab] OR "micro implant"[tiab] OR "micro implants"[tiab] OR "temporary anchorage device"[tiab] OR "temporary anchorage devices"[tiab] OR TAD[tiab] OR TADs[tiab] OR "skeletal anchorage"[tiab] OR "Orthodontic Anchorage Procedures"[Mesh])
AND
(palate[tiab] OR palatal[tiab] OR "anterior palate"[tiab] OR midpalatal[tiab] OR "mid-palatal"[tiab] OR "median palate"[tiab] OR "paramedian palate"[tiab] OR "T-zone"[tiab] OR "T zone"[tiab] OR "posterior palate"[tiab] OR PPSAIS[tiab] OR parapalatal[tiab] OR "para-palatal"[tiab])
AND
("primary stability"[tiab] OR "secondary stability"[tiab] OR "insertion torque"[tiab] OR "removal torque"[tiab] OR stability[tiab] OR survival[tiab] OR success[tiab] OR failure[tiab] OR mobility[tiab] OR loosening[tiab] OR fracture[tiab] OR "root contact"[tiab] OR complication*[tiab])

Block C — Miniscrew design, biomaterial and insertion mechanics (yield: 905)

(miniscrew*[tiab] OR "mini-screw"[tiab] OR "mini-screws"[tiab] OR miniimplant*[tiab] OR "mini-implant"[tiab] OR "mini-implants"[tiab] OR "mini implant"[tiab] OR "mini implants"[tiab] OR microimplant*[tiab] OR "micro-implant"[tiab] OR "micro-implants"[tiab] OR "micro implant"[tiab] OR "micro implants"[tiab] OR "temporary anchorage device"[tiab] OR "temporary anchorage devices"[tiab] OR TAD[tiab] OR TADs[tiab] OR "skeletal anchorage"[tiab] OR "Orthodontic Anchorage Procedures"[Mesh])
AND
(orthodontic*[tiab] OR orthodontics[tiab] OR anchorage[tiab])
AND
(design[tiab] OR diameter[tiab] OR length[tiab] OR thread*[tiab] OR "thread pitch"[tiab] OR "thread depth"[tiab] OR collar[tiab] OR neck[tiab] OR surface[tiab] OR titanium[tiab] OR alloy[tiab] OR "stainless steel"[tiab] OR biocompatibility[tiab] OR osseointegration[tiab] OR "self-drilling"[tiab] OR "self-tapping"[tiab] OR "pre-drilling"[tiab] OR predrilling[tiab] OR "pilot hole"[tiab])
AND
("primary stability"[tiab] OR "insertion torque"[tiab] OR "removal torque"[tiab] OR stability[tiab] OR survival[tiab] OR failure[tiab] OR fracture[tiab] OR bone[tiab] OR cortical[tiab])

Block D — MARPE / high-load skeletal anchorage (yield: 106)

(MARPE[tiab] OR "miniscrew-assisted rapid palatal expansion"[tiab] OR "miniscrew assisted rapid palatal expansion"[tiab] OR "microimplant-assisted rapid palatal expansion"[tiab] OR "microimplant assisted rapid palatal expansion"[tiab] OR "mini-implant assisted rapid palatal expansion"[tiab] OR "mini implant assisted rapid palatal expansion"[tiab] OR "maxillary skeletal expander"[tiab] OR MSE[tiab] OR "skeletal maxillary expansion"[tiab])
AND
(miniscrew*[tiab] OR "mini-screw"[tiab] OR "mini-screws"[tiab] OR miniimplant*[tiab] OR "mini-implant"[tiab] OR "mini-implants"[tiab] OR microimplant*[tiab] OR "micro-implant"[tiab] OR "micro-implants"[tiab] OR "temporary anchorage device"[tiab] OR "temporary anchorage devices"[tiab])
AND
(bicortical[tiab] OR monocortical[tiab] OR "cortical engagement"[tiab] OR stability[tiab] OR survival[tiab] OR failure[tiab] OR "bone thickness"[tiab] OR "insertion torque"[tiab] OR biomechanics[tiab] OR biomechanical[tiab] OR "finite element"[tiab] OR "force distribution"[tiab])

Block E — Digital planning and guided insertion (yield: 313)

(miniscrew*[tiab] OR "mini-screw"[tiab] OR "mini-screws"[tiab] OR miniimplant*[tiab] OR "mini-implant"[tiab] OR "mini-implants"[tiab] OR "mini implant"[tiab] OR "mini implants"[tiab] OR microimplant*[tiab] OR "micro-implant"[tiab] OR "micro-implants"[tiab] OR "temporary anchorage device"[tiab] OR "temporary anchorage devices"[tiab] OR TAD[tiab] OR TADs[tiab] OR "skeletal anchorage"[tiab])
AND
(palate[tiab] OR palatal[tiab] OR "anterior palate"[tiab] OR midpalatal[tiab] OR "mid-palatal"[tiab] OR "paramedian palate"[tiab] OR "posterior palate"[tiab] OR PPSAIS[tiab] OR MARPE[tiab])
AND
("digital planning"[tiab] OR "guided insertion"[tiab] OR "surgical guide"[tiab] OR "surgical guides"[tiab] OR "insertion guide"[tiab] OR "insertion guides"[tiab] OR navigation[tiab] OR "dynamic navigation"[tiab] OR CAD/CAM[tiab] OR CADCAM[tiab] OR STL[tiab] OR DICOM[tiab] OR CBCT[tiab] OR "static guide"[tiab] OR "static guides"[tiab] OR "3D printed"[tiab] OR "three-dimensional"[tiab] OR "computer-aided"[tiab])

Scopus

Block A — Palatal anatomical substrate (yield: 520)

TITLE-ABS-KEY ((miniscrew* OR "mini-screw*" OR miniimplant* OR "mini-implant*" OR "mini implant*" OR microimplant* OR "micro-implant*" OR "micro implant*" OR "temporary anchorage device*" OR TAD OR TADs OR "skeletal anchorage")
AND
(palate OR palatal OR "anterior palate" OR midpalatal OR "mid-palatal" OR "median palate" OR "paramedian palate" OR "T-zone" OR "T zone" OR "posterior palate" OR "palatal slope" OR "palatal vault" OR PPSAIS OR "palatal posterior supra-alveolar" OR parapalatal OR "para-palatal")
AND
("bone thickness" OR "cortical thickness" OR "cortical bone" OR "bone density" OR "bone depth" OR "bone volume" OR "mucosal thickness" OR "soft tissue thickness" OR CBCT OR "cone beam computed tomography" OR "computed tomography"))

Block B — Primary stability, survival and failure (yield: 388)

TITLE-ABS-KEY ((miniscrew* OR "mini-screw*" OR miniimplant* OR "mini-implant*" OR "mini implant*" OR microimplant* OR "micro-implant*" OR "micro implant*" OR "temporary anchorage device*" OR TAD OR TADs OR "skeletal anchorage")
AND
(palate OR palatal OR "anterior palate" OR midpalatal OR "mid-palatal" OR "median palate" OR "paramedian palate" OR "T-zone" OR "T zone" OR "posterior palate" OR PPSAIS OR parapalatal OR "para-palatal")
AND
("primary stability" OR "secondary stability" OR "insertion torque" OR "removal torque" OR stability OR survival OR success OR failure OR mobility OR loosening OR fracture OR "root contact" OR complication*))

Block C — Miniscrew design, biomaterial and insertion mechanics (yield: 1,610)

TITLE-ABS-KEY ((miniscrew* OR "mini-screw*" OR miniimplant* OR "mini-implant*" OR "mini implant*" OR microimplant* OR "micro-implant*" OR "micro implant*" OR "temporary anchorage device*" OR TAD OR TADs OR "skeletal anchorage")
AND
(orthodontic* OR orthodontics OR anchorage)
AND
(design OR diameter OR length OR thread* OR "thread pitch" OR "thread depth" OR collar OR neck OR surface OR titanium OR alloy OR "stainless steel" OR biocompatibility OR osseointegration OR "self-drilling" OR "self-tapping" OR "pre-drilling" OR predrilling OR "pilot hole")
AND
("primary stability" OR "insertion torque" OR "removal torque" OR stability OR survival OR failure OR fracture OR bone OR cortical))

Block D — MARPE / high-load skeletal anchorage (yield: 107)

TITLE-ABS-KEY ((MARPE OR "miniscrew-assisted rapid palatal expansion" OR "miniscrew assisted rapid palatal expansion" OR "microimplant-assisted rapid palatal expansion" OR "microimplant assisted rapid palatal expansion" OR "mini-implant assisted rapid palatal expansion" OR "mini implant assisted rapid palatal expansion" OR "maxillary skeletal expander" OR MSE OR "skeletal maxillary expansion")
AND
(miniscrew* OR "mini-screw*" OR miniimplant* OR "mini-implant*" OR microimplant* OR "micro-implant*" OR "temporary anchorage device*")
AND
(bicortical OR monocortical OR "cortical engagement" OR stability OR survival OR failure OR "bone thickness" OR "insertion torque" OR biomechanics OR biomechanical OR "finite element" OR "force distribution"))

Block E — Digital planning and guided insertion (yield: 73)

TITLE-ABS-KEY ((miniscrew* OR "mini-screw*" OR miniimplant* OR "mini-implant*" OR "mini implant*" OR microimplant* OR "micro-implant*" OR "temporary anchorage device*" OR TAD OR TADs OR "skeletal anchorage")
AND
(palate OR palatal OR "anterior palate" OR midpalatal OR "mid-palatal" OR "paramedian palate" OR "posterior palate" OR PPSAIS OR MARPE)
AND
("digital planning" OR "guided insertion" OR "surgical guide*" OR "insertion guide*" OR navigation OR "dynamic navigation" OR CAD/CAM OR CADCAM OR STL OR DICOM OR CBCT OR "static guide*" OR "3D printed" OR "three-dimensional" OR "computer-aided"))

Web of Science Core Collection

Block A — Palatal anatomical substrate (yield: 507)

TS=((miniscrew* OR "mini-screw*" OR miniimplant* OR "mini-implant*" OR "mini implant*" OR microimplant* OR "micro-implant*" OR "micro implant*" OR "temporary anchorage device*" OR TAD OR TADs OR "skeletal anchorage")
AND
(palate OR palatal OR "anterior palate" OR midpalatal OR "mid-palatal" OR "median palate" OR "paramedian palate" OR "T-zone" OR "T zone" OR "posterior palate" OR "palatal slope" OR "palatal vault" OR PPSAIS OR "palatal posterior supra-alveolar" OR parapalatal OR "para-palatal")
AND
("bone thickness" OR "cortical thickness" OR "cortical bone" OR "bone density" OR "bone depth" OR "bone volume" OR "mucosal thickness" OR "soft tissue thickness" OR CBCT OR "cone beam computed tomography" OR "computed tomography"))

Block B — Primary stability, survival and failure (yield: 489)

TS=((miniscrew* OR "mini-screw*" OR miniimplant* OR "mini-implant*" OR "mini implant*" OR microimplant* OR "micro-implant*" OR "micro implant*" OR "temporary anchorage device*" OR TAD OR TADs OR "skeletal anchorage")
AND
(palate OR palatal OR "anterior palate" OR midpalatal OR "mid-palatal" OR "median palate" OR "paramedian palate" OR "T-zone" OR "T zone" OR "posterior palate" OR PPSAIS OR parapalatal OR "para-palatal")
AND
("primary stability" OR "secondary stability" OR "insertion torque" OR "removal torque" OR stability OR survival OR success OR failure OR mobility OR loosening OR fracture OR "root contact" OR complication*))

Block C — Miniscrew design, biomaterial and insertion mechanics (yield: 1,269)

TS=((miniscrew* OR "mini-screw*" OR miniimplant* OR "mini-implant*" OR "mini implant*" OR microimplant* OR "micro-implant*" OR "micro implant*" OR "temporary anchorage device*" OR TAD OR TADs OR "skeletal anchorage")
AND
(orthodontic* OR orthodontics OR anchorage)
AND
(design OR diameter OR length OR thread* OR "thread pitch" OR "thread depth" OR collar OR neck OR surface OR titanium OR alloy OR "stainless steel" OR biocompatibility OR osseointegration OR "self-drilling" OR "self-tapping" OR "pre-drilling" OR predrilling OR "pilot hole")
AND
("primary stability" OR "insertion torque" OR "removal torque" OR stability OR survival OR failure OR fracture OR bone OR cortical))

Block D — MARPE / high-load skeletal anchorage (yield: 145)

TS=((MARPE OR "miniscrew-assisted rapid palatal expansion" OR "miniscrew assisted rapid palatal expansion" OR "microimplant-assisted rapid palatal expansion" OR "microimplant assisted rapid palatal expansion" OR "mini-implant assisted rapid palatal expansion" OR "mini implant assisted rapid palatal expansion" OR "maxillary skeletal expander" OR MSE OR "skeletal maxillary expansion")
AND
(miniscrew* OR "mini-screw*" OR miniimplant* OR "mini-implant*" OR microimplant* OR "micro-implant*" OR "temporary anchorage device*")
AND
(bicortical OR monocortical OR "cortical engagement" OR stability OR survival OR failure OR "bone thickness" OR "insertion torque" OR biomechanics OR biomechanical OR "finite element" OR "force distribution"))

Block E — Digital planning and guided insertion (yield: 386)

TS=((miniscrew* OR "mini-screw*" OR miniimplant* OR "mini-implant*" OR "mini implant*" OR microimplant* OR "micro-implant*" OR "temporary anchorage device*" OR TAD OR TADs OR "skeletal anchorage")<br>AND<br>(palate OR palatal OR "anterior palate" OR midpalatal OR "mid-palatal" OR "paramedian palate" OR "posterior palate" OR PPSAIS OR MARPE)<br>AND<br>("digital planning" OR "guided insertion" OR "surgical guide*" OR "insertion guide*" OR navigation OR "dynamic navigation" OR CAD/CAM OR CADCAM OR STL OR DICOM OR CBCT OR "static guide*" OR "3D printed" OR "three-dimensional" OR "computer-aided"))

Supplementary Table S12. PRISMA-ScR CHECKLIST.

SECTION / TOPIC	ITEM	PRISMA-ScR CHECKLIST ITEM	REPORTED ON PAGE(S) / STATUS
TITLE	1	**Title.** Identify the report as a scoping review.	p. 1
ABSTRACT	2	**Structured summary.** Provide a structured summary that includes, as applicable, background, objectives, eligibility criteria, sources of evidence, charting methods, results, and conclusions related to the review questions and objectives.	pp. 1-2
INTRODUCTION	3	**Rationale.** Describe the rationale for the review in the context of what is already known and explain why the review questions or objectives lend themselves to a scoping-review approach.	pp. 2-3
	4	**Objectives.** Provide an explicit statement of the questions and objectives being addressed, with reference to their key elements (e.g., population or participants, concept, and context).	pp. 3-4; Table 1
METHODS	5	**Protocol and registration.** Indicate whether a review protocol exists; state if and where it can be accessed; and, if available, provide registration information, including the registration number.	Section 2.1, p. 3; retrospectively registered on the Open Science Framework on 17 July 2026: https://osf.io/5bcxu
	6	**Eligibility criteria.** Specify characteristics of the sources of evidence used as eligibility criteria (e.g., years considered, language, and publication status), and provide a rationale.	p. 4; Supplementary Table S1
	7	**Information sources.** Describe all information sources in the search, as well as the date the most recent search was executed.	Section 2.2, p. 4; final search date: 26 May 2026
	8	**Search.** Present the full electronic search strategy for at least one database, including any limits used, so that it could be repeated.	Section 2.2, p. 4; Supplementary Table S11
	9	**Selection of sources of evidence.** State the process for selecting sources of evidence (i.e., screening and eligibility) included in the scoping review.	Sections 2.2-2.3, pp. 4; Supplementary Table S1
	10	**Data charting process.** Describe the methods of charting data from the included sources of evidence, including whether charting was independent or in duplicate, and any processes for obtaining or confirming data.	Sections 2.3-2.4, p. 4-5
	11	**Data items.** List and define all variables for which data were sought and any assumptions and simplifications made.	Section 2.4, pp. 4-5; Supplementary Tables S3-S10; Supplementary Data S2
	12	**Critical appraisal of individual sources of evidence.** If done, provide a rationale for conducting a critical appraisal, describe the methods used, and explain how the information was used in any synthesis.	Not undertaken; not applicable to the evidence-mapping objective
	13	**Synthesis of results.** Describe the methods of handling and summarizing the data that were charted.	Section 2.5, p. 5; Supplementary Tables S2-S5
RESULTS	14	**Selection of sources of evidence.** Give numbers of sources screened, assessed for eligibility, and included in the review, with reasons for exclusions at each stage, ideally using a flow diagram.	Section 3.1, p. 6; Figure 1
	15	**Characteristics of sources of evidence.** For each source of evidence, present characteristics for which data were charted and provide the citations.	Results, pp. 6–25; Tables 2–10; Supplementary Tables S3–S10; Supplementary Data S2
	16	**Critical appraisal within sources of evidence.** If done, present data on critical appraisal of included sources of evidence.	Not undertaken; not applicable
	17	**Results of individual sources of evidence.** For each included source of evidence, present the relevant data that were charted and that relate to the review questions and objectives.	Results, pp. 6–25; Tables 2–10; Supplementary Tables S3–S10; Supplementary Data S2
	18	**Synthesis of results.** Summarize and/or present the charting results as they relate to the review questions and objectives.	Results, pp. 6–25; Tables 2–10; Figures 2–5
DISCUSSION	19	**Summary of evidence.** Summarize the main results, link them to the review questions and objectives, and consider their relevance to key groups.	Discussion, pp. 25–26
	20	**Limitations.** Discuss the limitations of the scoping-review process.	Section 4.6, p. 26
	21	**Conclusions.** Provide a general interpretation of the results with respect to the review questions and objectives, as well as potential implications and/or next steps.	Section 5, p. 26
FUNDING	22	**Funding.** Describe sources of funding for the included sources of evidence and for the scoping review, and describe the role of the funders of the scoping review.	p. 27; Funding statement. Funding of individual sources of evidence was not systematically charted.

Abbreviations: PRISMA-ScR, Preferred Reporting Items for Systematic Reviews and Meta-Analyses Extension for Scoping Reviews.

Checklist source: Tricco AC, Lillie E, Zarin W, et al. PRISMA Extension for Scoping Reviews (PRISMA-ScR): Checklist and Explanation. Ann Intern Med. 2018;169:467-473. doi:10.7326/M18-0850.
